# Ionizable Lipid‐Dependent Optimization of Steroid Lipid Nanoparticles With Tunable Immunomodulatory Properties

**DOI:** 10.1002/adma.74301

**Published:** 2026-08-06

**Authors:** Ajay S. Thatte, Benjamin E. Nachod, Julia Baena, Jenna Muscat‐Rivera, Lesley Chaboub, Hannah C. Safford, Hannah C. Geisler, Hannah M. Yamagata, Melgious J. Y. Ang, Michael Kegel, Alexandre Poirier, Elaine R. Tong, Zhe Zhong, Qiangqiang Shi, Jinjin Wang, Elisa Battistini, Ye Zeng, Alex G. Hamilton, Kelsey L. Swingle, Drew Weissman, Jilian R. Melamed, Michael J. Mitchell

**Affiliations:** ^1^ Department of Bioengineering University of Pennsylvania Philadelphia Pennsylvania USA; ^2^ Division of Infectious Disease Perelman School of Medicine University of Pennsylvania Philadelphia Pennsylvania USA; ^3^ Penn Institute for RNA Innovation Perelman School of Medicine University of Pennsylvania Philadelphia Pennsylvania USA; ^4^ Institute for Immunology Perelman School of Medicine University of Pennsylvania Philadelphia Pennsylvania USA; ^5^ Cardiovascular Institute Perelman School of Medicine University of Pennsylvania Philadelphia Pennsylvania USA; ^6^ Institute for Regenerative Medicine Perelman School of Medicine University of Pennsylvania Philadelphia Pennsylvania USA; ^7^ Abramson Cancer Center Perelman School of Medicine University of Pennsylvania Philadelphia Pennsylvania USA; ^8^ Center for Precision Engineering for Health University of Pennsylvania Philadelphia Pennsylvania USA

**Keywords:** autoimmune disease, immunomodulatory materials, lipid nanoparticles, nanomaterials design, nanotechnology, nucleic acid delivery

## Abstract

Lipid nanoparticles (LNPs) are a leading platform for nucleic acid delivery, yet their intrinsic adjuvanticity poses a significant materials design challenge for applications requiring immunological quiescence. Here, we report a modular engineering strategy that incorporates FDA‐approved corticosteroids into LNP formulations, creating a new class of steroid LNPs with tunable anti‐inflammatory properties. Through systematic screening of steroid and cholesterol substitution ratios, we establish structure–property relationships governing mRNA encapsulation efficiency, physicochemical characteristics, and inflammation suppression. Triamcinolone (TRI) emerges as our lead steroid, with 50% cholesterol substitution in SM‐102 LNPs preserving physicochemical characteristics. Importantly, we show that optimal substitution ratios are ionizable lipid‐dependent—80% for MC3 and 50% for SM‐102 and ALC‐0315—revealing fundamental design principles for these dual‐functional LNPs. In an endotoxemia mouse model, TRI LNPs administered intramuscularly maintain mRNA delivery efficacy while reducing inflammatory cytokines by ∼4‐fold compared to SM‐102 LNPs. In a multiple sclerosis mouse model, TRI LNPs delivering therapeutic mRNA promote antigen‐specific tolerance in spinal cord tissue and protect against paralysis. Compared to SM‐102 LNPs, TRI LNPs reduce inflammatory cytokines by ∼3‐fold and prolong protection against paralysis. Together, our work introduces a generalizable materials design strategy for engineering LNPs with tunable immunomodulatory properties to expand their therapeutic utility.

## Graphical abstract

A novel engineering strategy that establishes material design principles for incorporating anti‐inflammatory steroids into lipid nanoparticles to reduce LNP‐induced inflammation while retaining mRNA delivery. Results are validated in vitro and in three animal models of inflammation and autoimmunity in vivo.

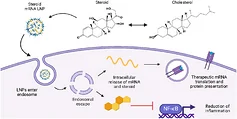

## 1 Introduction

Lipid nanoparticles (LNPs) have emerged as a leading delivery platform for nucleic acids such as messenger RNA (mRNA), exemplified by the clinical success of mRNA LNP vaccines during the COVID‐19 pandemic [1, 2]. Their four‐component architecture—ionizable lipid, helper phospholipid, cholesterol, and polyethylene glycol (PEG)‐lipid—enables precise control over nanoparticle self‐assembly, cargo encapsulation, biodistribution, and endosomal escape [3, 4]. As such, LNPs have become an exceptionally versatile delivery platform, with studies demonstrating their use in applications ranging from infectious disease vaccines to cancer therapy and in vivo CAR T cell engineering for cancer immunotherapy [5, 6, 7, 8, 9].

Despite this versatility, a fundamental materials challenge persists. LNP delivery efficiency is dictated primarily by lipid composition and architecture, in particular the ionizable lipid, which defines the chemical identity of the LNP and drives endosomal disruption and cytosolic release of nucleic acid cargo [3, 5, 10]. Potent ionizable lipids, however, also activate pattern recognition receptors (PRRs) such as toll‐like receptor 4 (TLR4), triggering NF‐κB–mediated acute inflammation and worsening any pre‐existing inflammatory state [11, 12, 13, 14]. Although such adjuvanticity can be beneficial in vaccines and cancer immunotherapy, it limits the use of LNPs in settings that require immunological quiescence, including autoimmune diseases such as multiple sclerosis [15, 16, 17]. Addressing this design challenge therefore requires strategies that maintain mRNA delivery efficacy while incorporating anti‐inflammatory functionality directly into the nanoparticle architecture.

Several approaches to limit LNP adjuvanticity have been explored, including dampening endolysosomal damage with galectin inhibitors or altering ionizable lipid chemistry [13, 14]. However, these strategies have notable limitations: the former relies on systemic pre‐treatment with small‐molecule inhibitors that broadly hinder necessary galectin function, and the latter modifies the ionizable lipid itself, which can compromise delivery efficiency and can demand laborious synthesis and purification [10, 13, 18]. We hypothesized that a modular, structure‐guided strategy that provides LNPs with localized anti‐inflammatory activity while preserving their delivery performance could expand their utility beyond vaccines and cancer.

Here, we present such a strategy by engineering LNPs with corticosteroids to create a new class of steroid LNPs with tunable anti‐inflammatory properties. Through systematic screening of six FDA‐approved steroids and optimization of cholesterol substitution ratios, we identify key structure–property relationships governing mRNA encapsulation, physicochemical characteristics, and inflammation suppression. We discover that optimal substitution of Triamcinolone (TRI), our lead steroid, depends on the ionizable lipid identity: SM‐102 and ALC‐0315 formulations require a 50% substitution ratio, whereas DLin‐MC3‐DMA requires an 80% substitution ratio. Upon intramuscular (i.m.) administration in an endotoxemia mouse model, optimized SM‐102 LNPs with 50% TRI substitution (TRI LNPs) retain robust mRNA delivery to lymph nodes while reducing inflammatory serum cytokine levels by ∼4‐fold and shifting antigen‐specific T cell phenotypes in draining lymph nodes toward a less inflammatory, more tolerogenic state compared to SM‐102 LNPs. Notably, in a multiple sclerosis mouse model, i.m. administration of TRI LNPs also protects against paralysis by inducing antigen‐specific tolerance and reducing inflammatory T cell infiltration into the spinal cord (Figure 1).

**FIGURE 1:**
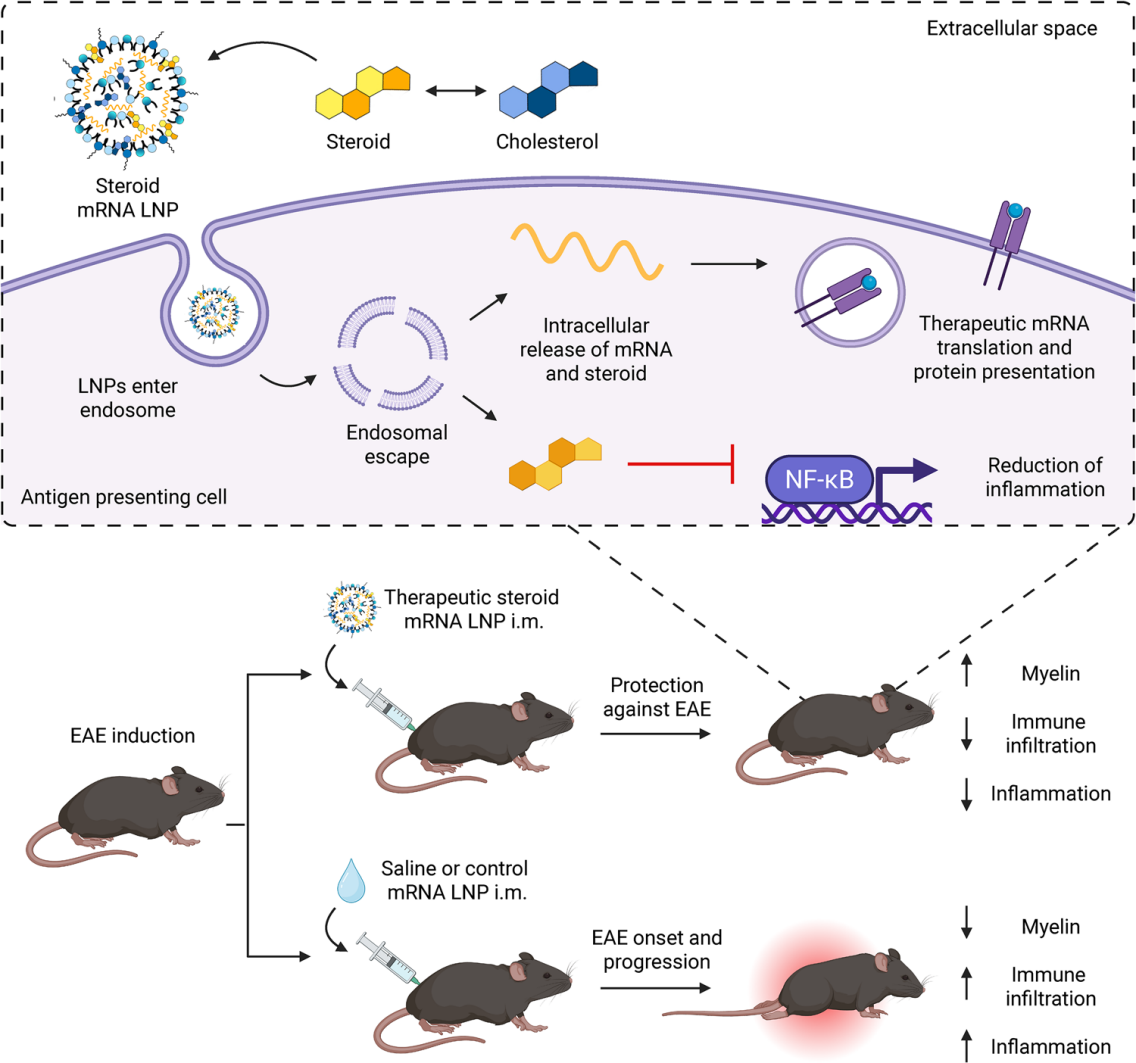
Schematic overview of steroid LNPs delivering mRNA, reducing inflammation and preventing autoimmune disease. Steroid LNPs are formulated by substituting a portion of the cholesterol component of the LNP with steroid. Upon endocytosis by antigen presenting cells (APCs), the LNPs escape the endosome and simultaneously deliver mRNA and steroid intracellularly. The therapeutic mRNA is translated and presented on the surface of the APCs while the steroid prevents activation of intracellular mechanisms of inflammation (e.g., NF‐κB activation). Experimental autoimmune encephalomyelitis (EAE)‐induced mice treated with either saline (no treatment), control TRI LNPs encapsulating Fluc mRNA or control SM‐102 LNPs encapsulating therapeutic MOG mRNA do not durably prevent disease onset and progression. However, steroid LNPs encapsulating therapeutic MOG mRNA strongly protect against disease and prevent immune infiltration into spinal cord tissue, preventing disease onset and progression.

Together, our findings establish a generalizable materials design strategy that mitigates LNP‐induced inflammation while preserving mRNA delivery efficiency, thereby enhancing the safety of mRNA LNPs and broadening their utility in inflammatory and autoimmune disease.

## 2 Results

### 2.1 Rational Design and Structure–Property Screening of Steroid LNPs

We first investigated whether incorporating corticosteroids (hereafter referred to as steroids) into LNP formulations could reduce LNP adjuvanticity. For this preliminary study, we used dexamethasone (DEX) as a representative steroid as DEX has previously been incorporated into LNPs (Figure 2a) [19]. Given their structural similarity, we substituted the cholesterol component to incorporate steroids into our LNPs. Specifically, we first incorporated DEX into the organic phase by substituting either 0%, 10%, or 50% of the cholesterol component and formulated SM‐102 LNPs encapsulating Firefly luciferase (Fluc) mRNA using microfluidic mixing (Figure 2b) [20]. To model inflammation, we first pre‐treated RAW‐Blue NF‐κB reporter cells with lipopolysaccharide (LPS) and then treated with either free DEX or 0%, 10%, and 50% DEX LNPs [21, 22]. Both LNP formulations with DEX (10% and 50%) suppressed NF‐κB activation comparably to free DEX, while SM‐102 LNPs without DEX (0%) exacerbated NF‐κB activation (Figure S1). These results confirmed that steroids incorporated into LNPs retained their anti‐inflammatory function and reduced LNP adjuvanticity.

**FIGURE 2:**
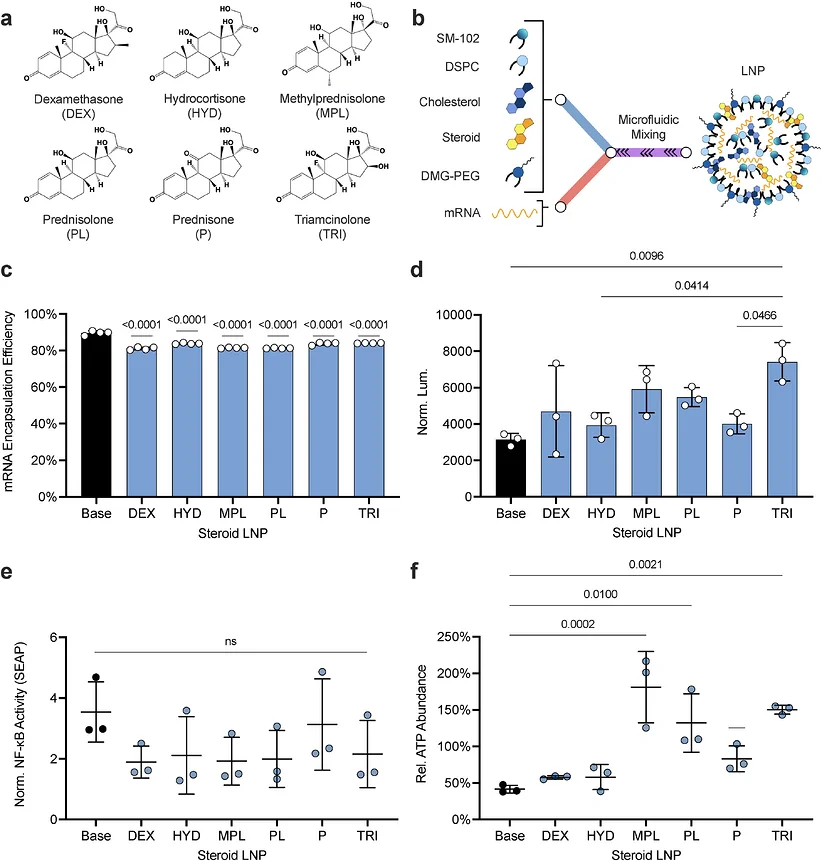
In vitro screening of steroid LNP library to establish design rules governing mRNA delivery, inflammation and cell health. (a) Chemical structures of steroids screened. (b) Schematic of LNP formulation depicting lipid and mRNA components being combined using microfluidic mixing. (c) Quantification of mRNA encapsulation efficiency of steroid LNP library. (d) Quantification of Firefly luciferase (Fluc) mRNA delivery, (e) corresponding NF‐κB activity, and (f) relative ATP abundance in LPS‐treated RAW‐Blue cells upon treatment with steroid LNP library at a dose of 100 ng/100,000 cells. All samples for (d–f) were normalized to a sample with no treatment. Data represent mean ± SD for *n* = 3 biological replicates. Statistical differences were determined by two‐sided, one‐way ANOVA with post hoc Tukey's correction. Full list of multiple comparisons and respective *p* values for (f) are listed in Table S2.

Having established the functionality of DEX LNPs, we next systematically screened six different FDA‐approved steroids incorporated into SM‐102 LNPs and assessed their ability to encapsulate and deliver mRNA while retaining their anti‐inflammatory activity. Specifically, we chose DEX, hydrocortisone (HYD), methylprednisolone (MPL), prednisolone (PL), prednisone (P), and TRI (Figure 2a). P was included as a negative control, as it is a structurally similar but inactive prodrug of PL and should not exhibit anti‐inflammatory activity [23]. To maintain consistent lipid composition across formulations, each steroid was substituted for 50% of the cholesterol component, while keeping total lipid levels constant. All six steroids were successfully incorporated into SM‐102 LNPs and retained key physicochemical properties, including mRNA encapsulation efficiency >80%, sizes between 75‐90 nm, and polydispersity index (PDI) values between 0.05‐0.15 (Figure 2b,c, Table S1) [24]. This was expected as all of the steroids comprise hydrocarbon rings, which exhibit structural similarity to cholesterol and maintain hydrophobic interactions to preserve LNP architecture [25].

We then evaluated the functional performance of these steroid LNPs in inflamed RAW‐Blue cells. All formulations successfully delivered Fluc mRNA under inflammatory conditions, with TRI LNPs showing significant enhancement in mRNA delivery compared to control Base LNPs with no steroid (Figure 2d). Additionally, while not significant at the selected treatment dose, all LNPs containing active steroids reduced NF‐κB activation compared to Base LNPs, whereas P LNPs containing the inactive steroid did not (Figure 2e). We also assessed relative ATP levels upon LNP treatment as a measurement of cell viability. MPL, PL, and TRI LNPs rescued ATP levels significantly compared to Base LNPs, whereas DEX, HYD, and P LNPs did not (Figure 2f, Table S2).

Our steroid LNP screen showed several interesting emerging structure‐property relationships. Among the active steroids, HYD performed poorly in terms of mRNA delivery and cell viability and was the only steroid lacking a C1─C2 double bond, possessing only the C4─C5 double bond (Figure S2). In contrast, MPL, PL, and TRI—all containing two double bonds—performed well, with TRI exhibiting the greatest enhancement in mRNA delivery and the strongest preservation of cell viability. Notably, this trend did not extend to DEX, despite its immense structural similarity to TRI. Although DEX and TRI both contain a fluoro substituent and C1─C2 and C4─C5 double bonds, they differ in their 16‐position modifications: DEX carries a methyl (16⍺‐CH_3_) whereas TRI carries a hydroxyl (16⍺‐OH) group. This 16α‐OH substitution may underlie the improved cellular viability observed with TRI [26]. Taken together, LNPs incorporating TRI emerged as the lead steroid LNP, resulting in superior mRNA delivery, suppressing NF‐κB activity, and maintaining cellular ATP levels and overall cell health in vitro.

### 2.2 Ionizable Lipid‐Dependent Structure–Property Relationships Govern Optimal Steroid Incorporation

After identifying TRI as the lead steroid, we next aimed to establish design rules for LNP engineering with TRI. We began by systematically optimizing the ratio of TRI within the SM‐102 LNP formulation. A stepwise substitution of TRI showed that replacing more than 50% of the cholesterol component led to a sharp decline in mRNA encapsulation efficiency from 80% to 53% (Figure 3a). Although structurally similar to cholesterol, TRI contains several polar functional groups, including C11β‐OH, C16α‐OH, C17α‐OH, C21‐OH, C3 ketone, C20 ketone, and C9⍺‐fluorine, making it more hydrophilic than cholesterol and potentially disrupting LNP structure at higher substitution ratios. Based on these findings, we selected four representative formulations for further evaluation: Base (0%), 30%, 50%, and 80% TRI‐substituted LNPs. All formulations maintained sizes between 70‐95 nm and PDI values between 0.04‐0.21 (Figure 3b, Table S3). Both the 50% and 80% TRI‐substituted LNPs significantly enhanced mRNA delivery in inflamed RAW‐Blue cells in a dose‐dependent manner (Figure 3c), while 30%, 50%, and 80% substitutions all led to significant reductions in NF‐κB activity (Figure 3d).

**FIGURE 3:**
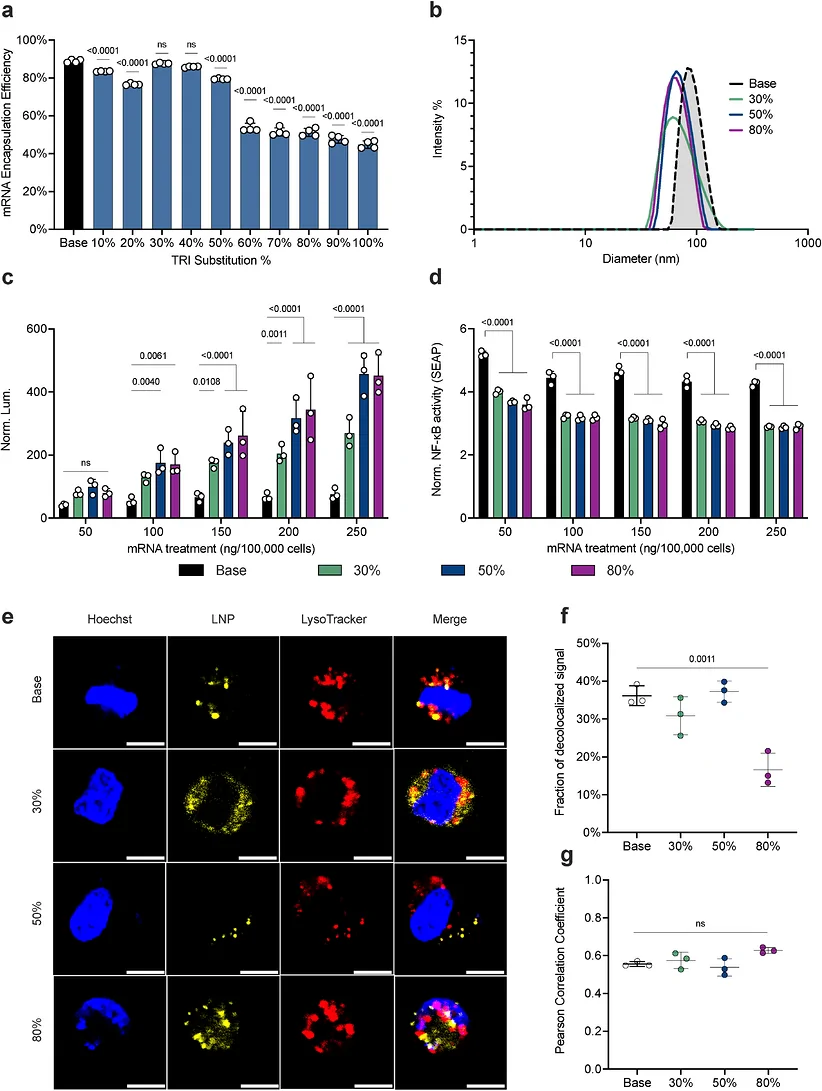
Systematic substitution of triamcinolone shows that design rules governing mRNA delivery, inflammation and LNP uptake are ionizable lipid‐dependent. (a) Quantification of mRNA encapsulation efficiency in SM‐102 LNPs with a step‐wise substitution of cholesterol for triamcinolone (TRI). (b) Representative hydrodynamic diameter intensity distribution of Base (0%), 30%, 50%, and 80% TRI substituted LNPs. (c) Quantification of Fluc mRNA delivery and (d) corresponding NF‐κB activation in inflamed RAW‐Blue cells of the four LNP formulations. Data represent mean + SD for *n* = 3 biological replicates. Statistical differences were determined by two‐sided, one‐way ANOVA with post hoc Tukey's correction. (e) Confocal microscopy images demonstrating DiI‐labeled LNP uptake in inflamed RAW 264.7 cells 4 h post‐treatment. Scale bar: 10 µm. (f) Quantification of % decolocalized signal between DiI‐labeled LNPs and Lysotracker‐labeled endosomes and (g) corresponding Pearson correlation coefficients of the 4 LNP formulations. Data represent mean ± SD for *n* = 3 fields of view and *n* = 5 cells in each field of view. Statistical differences were determined by two‐sided, one‐way ANOVA with post hoc Tukey's correction.

We next examined the uptake kinetics for all four DiI dye‐labeled LNPs in inflamed RAW 264.7 and DC2.4 cells via flow cytometric analysis. We found that all three TRI‐substituted LNPs were taken up at higher levels than the Base LNP in both cell lines at 1 and 4 h post‐treatment (Figure S3). We further examined LNP localization within cellular endosomes of inflamed RAW 264.7 cells using confocal microscopy. We observed that the 30% and 50% formulations showed similar levels of endosomal decolocalization compared to the Base formulation, with approximately 30%–40% of LNP signal not associated with endosomal signal (Figures 3e,f and S4). This was further supported by comparable Pearson correlation coefficients for the LNPs (Figure 3g). Taken together, these results establish the design rule that in the SM‐102 LNP formulation, 50% TRI substitution is optimal as it achieves the highest level of steroid incorporation without drastically compromising mRNA encapsulation while also potentiating effective mRNA delivery, LNP uptake and inflammation reduction.

To probe the extent to which our 50% TRI‐substituted SM‐102 LNPs (TRI LNPs) were able to reduce inflammation, we benchmarked them against several gold‐standard LNP formulations, including DLin‐MC3‐DMA (MC3), C12‐200, 4A3‐SC8, and SM‐102 and evaluated expression of downstream NF‐κB targets genes. RT‐qPCR analysis showed that, with LPS pre‐treatment, TRI LNPs induced significantly lower CCL2 expression than C12‐200 and SM‐102 without significantly exceeding the untreated control (Figure S5a). ELISA analysis further demonstrated that only TRI LNPs significantly reduced IL‐6 levels compared to C12‐200, 4A3‐SC8, and SM‐102, confirming that a 50% incorporation of TRI in SM‐102 LNPs was sufficient to reduce LNP‐induced inflammation (Figure S5b).

We next sought to evaluate the stability of our lead steroid LNP, for which we measured LNP size, PDI and mRNA encapsulation efficiency of both Base and TRI LNPs over 28 days with storage at 4°C. We observed a gradual increase in size for both Base and TRI LNPs, with TRI LNPs being significantly smaller than Base LNPs on days 3, 11, and 14 (Figure S6). Compared to their respective sizes on day 1 (98.8 nm for Base, 97.8 nm for TRI), both LNPs demonstrated significant increases in size on day 14 (117.4 nm for Base, 110.4 nm for TRI), which persisted to day 28 (122.5 nm for Base, 122.5 nm for TRI) (Table S4). As expected, PDI values also significantly increased on day 14 and day 28 for both LNPs (Figure S6, Table S5). mRNA encapsulation efficiency for both LNPs were consistently between 80%–90% for the first 7 days. However, we observed a slight decrease on day 11 and day 14, with TRI LNPs demonstrating a more significant decrease from 84.3% to 64.1% on day 14 (Figure S6, Table S6). Interestingly, day 28 measurements showed that both LNPs had rescued encapsulation efficiencies of 85.4% for Base LNP and 85.8% for TRI LNP. Together, our results indicate that both Base and TRI LNPs demonstrated desirable physicochemical characteristics for only 11 days at 4°C post‐formulation, suggesting than incorporation of TRI into LNPs according to our design rule did not drastically alter their stability.

We further investigated whether the design rule we found in the SM‐102 LNP was applicable across other LNP formulations with different ionizable lipid structures and chemical identities. To answer this question, we formulated LNPs using two other industry‐standard ionizable lipids—DLin‐MC3‐DMA (MC3) and ALC‐0315—with 0%, 30%, 50%, and 80% TRI substitutions and evaluated physicochemical characteristics as well as mRNA delivery and NF‐κB activation in inflamed RAW‐Blue cells. All LNPs exhibited sizes and PDI values within previously reported ranges (Tables S7 and S8) [24, 27]. Interestingly, MC3 LNPs with 80% TRI substitution achieved the highest mRNA delivery and greatest reduction in NF‐κB activity (Figure S7a,b). This may be due to the unsaturated linoleyl tails within the MC3 structure, allowing for less tightly packed LNPs and enabling more steroid incorporation [27, 28]. In contrast, ALC‐0315 LNPs with 50% TRI substitution resulted in the highest mRNA delivery, with all three substitution ratios reducing NF‐κB activity at higher treatment doses (Figure S7c,d). However, 80% TRI substitution in ALC‐0315 LNPs led to poor mRNA delivery. This may be due to ALC‐0315 having bulkier, saturated branched groups, allowing for more tightly packed LNPs but also reducing the optimal amount of steroid incorporated [24]. These findings demonstrate that the design rules for optimal TRI substitution are ionizable lipid‐dependent, with unsaturated ionizable lipids tolerating higher steroid ratios and branched, saturated ionizable lipids tolerating lower steroid ratios.

### 2.3 Performance Validation of Optimized TRI LNPs in a Mouse Model of Endotoxemia

To validate that TRI incorporation reduces LNP adjuvanticity and subsequent inflammation without compromising mRNA delivery in vivo, we evaluated the biodistribution and functional mRNA delivery of SM‐102 LNPs and structure–property‐optimized SM‐102 LNPs with 50% TRI substitution (TRI LNPs). We administered DiD dye‐labeled SM‐102 and TRI LNPs encapsulating Fluc mRNA intramuscularly (i.m.) in a mouse model of LPS‐induced endotoxemia (Figure 4a). 4 h post‐treatment, optimized TRI LNPs showed comparable accumulation to SM‐102 LNPs in both iliac and inguinal lymph nodes, with negligible accumulation in the lungs, liver, and spleen (Figure S8). Furthermore, TRI LNPs demonstrated comparable Fluc mRNA delivery to both sets of lymph nodes compared to SM‐102 LNPs (Figure 4b–d). As expected upon i.m. administration, neither LNP resulted in productive mRNA delivery to the lungs and SM‐102 LNPs resulted in modestly higher delivery to the liver than TRI LNPs (Figure S9a,b). Both LNPs potentiated effective mRNA delivery to the spleen, potentially due to increased immune trafficking in an inflamed environment (Figure S9c) [29].

**FIGURE 4:**
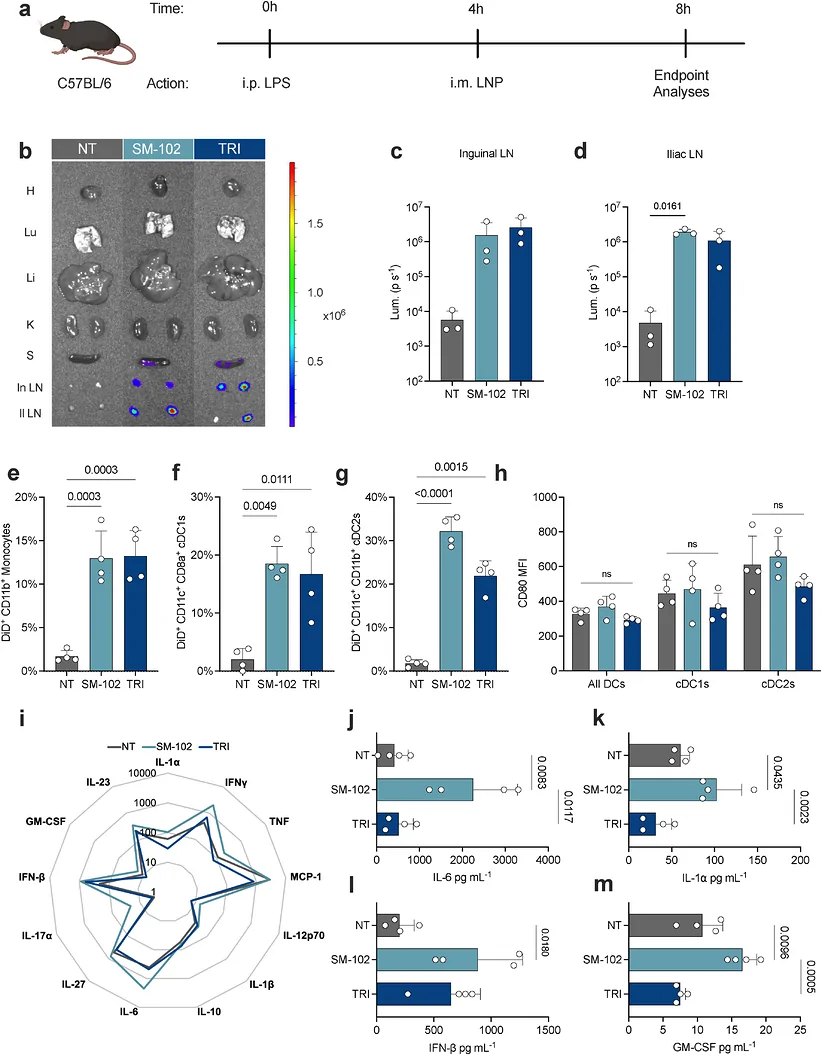
mRNA delivery, biodistribution and cytokine response of TRI LNPs in endotoxemia mouse model. (a) Experimental design and timeline of LNP treatment in endotoxemia mouse model. 0.1 mg/kg LPS was administered intraperitoneally (i.p.) to induce inflammation. At 4 h post‐LPS, a 5 µg dose of either SM‐102 or TRI LNPs encapsulating Fluc mRNA was administered i.m. Mice that received no LNP treatment (NT) served as a control group. At 4 h post‐LNP treatment, imaging, flow cytometry, and cytokine analyses were performed. (b) Representative IVIS image of luminescence in major organs and corresponding quantification in (c) inguinal lymph nodes and (d) iliac lymph nodes. Data represent mean + SD for *n* = 3 biological replicates. Statistical differences were determined by one‐way ANOVA with post hoc Tukey's correction. H, heart; Lu, lungs; Li, liver; K, kidneys; S, spleen; In LN, inguinal lymph nodes; Il LN, iliac lymph nodes. (e–g) Quantification of uptake of DiD‐labeled LNPs in (e) CD11b^+^ monocytes, (f) CD8a^+^ CD11c^+^ conventional dendritic cells (cDC1s), and (g) CD11b^+^ CD11c^+^ dendritic cells (cDC2s). (h) Quantification of CD80 expression on DC subsets. (i) Multiplexed analysis of inflammatory cytokines 4 h post‐LNP treatment. (j–m) Individual cytokine levels of (j) IL‐6, (k) IL‐1⍺, (l) IFN‐β, and (m) GM‐CSF. Data represent mean + SD for *n* = 4 biological replicates. Statistical differences were determined by two‐sided, one‐way ANOVA with post hoc Tukey's correction.

At the cellular level, both SM‐102 and TRI LNPs were comparably and predominantly taken up by phagocytic antigen‐presenting cells (APCs), including monocytes, cDC1, and cDC2 cells, with negligible uptake by lymphocytes (Figure 4e–g; Figures S10 and S11). Interestingly, while SM‐102 LNPs modestly increased expression of the DC activation marker CD80 on both DC subsets from baseline, TRI LNPs did not (Figure 4h). Together, our data suggests that TRI LNPs are effectively taken up by APCs and deliver mRNA.

We further assessed systemic cytokine levels to evaluate the acute inflammatory response caused by LNP adjuvanticity. In healthy mice (no LPS), i.m. administration of both SM‐102 and TRI LNPs led to no significant changes in levels of cytokines such as IFN‐β, GM‐CSF, IL‐1⍺, and IL‐10, with other cytokines being below the limit of detection (Figure S12). In mice with endotoxemia, however, SM‐102 LNPs exacerbated pre‐existing inflammation by significantly increasing several inflammatory cytokines, notably including IL‐6, IL‐1⍺, IFN‐β, and GM‐CSF (Figures 4i–m and S13). While such effects have previously been reported for intravenous LNP administration, this result highlights the potential of LNPs to exacerbate pre‐existing inflammation even after i.m. administration [11]. In contrast, TRI LNPs consistently induced lower levels of all measured inflammatory cytokines, in some cases reducing levels even below those of the no treatment (NT) control (Figures 4i–m and S13). Together, these results demonstrate that our optimized TRI LNPs achieve mRNA delivery comparable to SM‐102 LNPs while mitigating acute LNP‐induced inflammation, validating our materials design strategy and highlighting the safety advantage of TRI LNPs over standard LNPs in inflamed environments.

### 2.4 Functional Validation of Optimized TRI LNPs in an Inflamed OT‐II Adoptive Transfer Mouse Model

Having confirmed that TRI LNPs can deliver mRNA to APCs without triggering excessive immune activation, we next evaluated their ability to functionally induce antigen‐specific T cell tolerance. To validate this, we employed an adoptive transfer model using OT‐II cells, which possess T cell receptors (TCRs) specific to ovalbumin (OVA) [30]. Specifically, we isolated OT‐II CD4^+^ T cells from donor OT‐II mice on a C57BL/6 background, fluorescently labeled them, and intravenously transplanted them into recipient C57BL/6 mice. These transplanted cells served as a model for pre‐existing antigen‐specific T cells, allowing us to evaluate their phenotype following treatment with LNPs encapsulating OVA‐encoding mRNA. To mimic an inflammatory environment, we also administered LPS i.p. We then treated the recipient mice with SM‐102 or TRI LNPs encapsulating OVA‐encoding mRNA via i.m. administration and assessed T cell responses in the OT‐II population upon restimulation with anti‐CD28 and OVA_323‐339_ peptide 2 days later (Figures 5a and S14).

**FIGURE 5:**
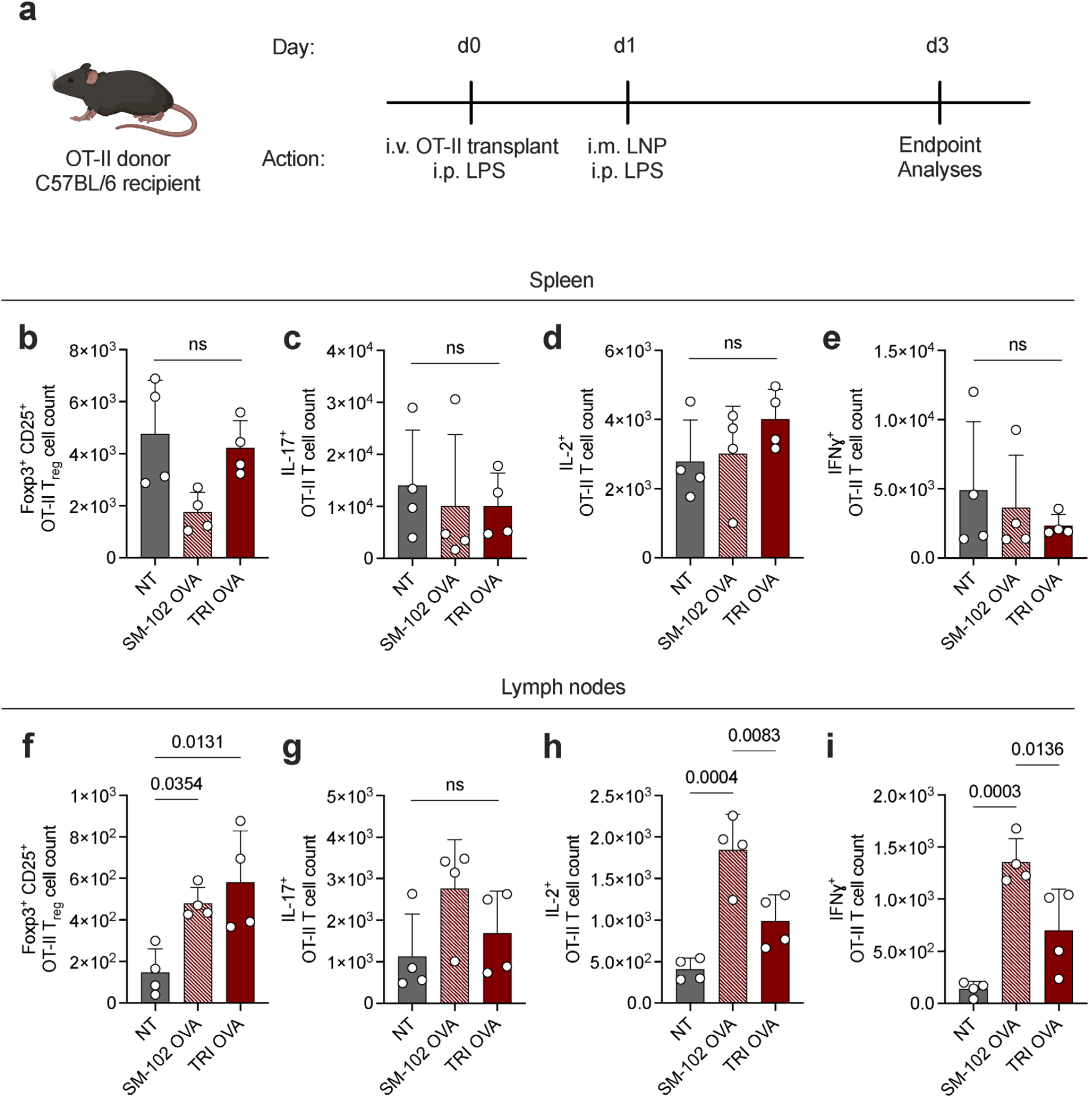
TRI LNPs skew antigen‐specific T cells toward a less inflammatory phenotype. (a) Experimental design and timeline of LNP treatment in inflamed OT‐II adoptive transfer model. On day 0, 1 million CellTrace Violet (CTV)‐labeled donor OT‐II cells were intravenously transplanted into recipient C57BL/6 mice. 0.1 mg/kg LPS was administered i.p. to induce inflammation. On day 1, a 5 µg dose of either SM‐102 or TRI LNPs encapsulating OVA mRNA was administered i.m. Mice that received no LNP treatment (NT) served as a control group. A second injection of LPS was also administered i.p. On day 3, mice were sacrificed and lymph nodes were harvested and processed for T cell restimulation and phenotype analysis. (b–e) Quantification of CTV‐labeled OT‐II T cell counts in the spleen that are (b) Foxp3^+^ CD25^+^, (c) IL‐17^+^, (d) IL‐2^+^, and (e) IFNɣ^+^. (f–i) Quantification of CTV‐labeled OT‐II T cell counts in the lymph nodes that are (b) Foxp3^+^ CD25^+^, (c) IL‐17^+^, (d) IL‐2^+^, and (e) IFNɣ^+^. Data represent mean + SD for *n* = 4 biological replicates. Statistical differences were determined by two‐sided, one‐way ANOVA with post hoc Tukey's correction.

In the spleen, TRI LNP treatment led to an increase in regulatory T (*T*_reg_) cells and a modest increase in IL‐2^+^ T cells along with a reduction in IL‐17^+^ and IFNɣ^+^ T cells in the OT‐II population, compared to the SM‐102 control (Figure 5b–e). This indicated that optimized TRI LNPs were able to functionally control and reduce the inflammatory skew of antigen‐specific T cell phenotypes more effectively than SM‐102 LNPs [31]. However, due to the early time point of 2 days post‐treatment, stronger changes in T cell phenotypes were observed in the draining lymph nodes than in the spleen. In the lymph nodes, both TRI LNPs and SM‐102 LNPs induced a significant increase in T_reg_ cell numbers relative to the NT group (Figure 5f). However, SM‐102 LNPs also increased IL‐17^+^, IL‐2^+^, and IFNɣ^+^ T cell numbers compared to the NT group, suggesting a strong inflammatory and potential Th1/Th17 skew in T cell phenotype (Figure 5g–i). This may undermine the tolerogenic function of T_reg_ cells as T_reg_ cells have been shown to respond to cues from Th1/Th17‐polarized T cells and can downregulate Foxp3 expression, impairing their suppressive capabilities [32, 33, 34]. In stark contrast, TRI LNPs led to a decrease in IL‐17^+^ T cells and a significant decrease in IL‐2^+^ and IFNɣ^+^ T cells compared to SM‐102 (Figure 5g–i). These results suggest that our optimized TRI LNPs preferentially drive antigen‐specific T cells toward a less inflammatory, more tolerogenic phenotype in the lymph nodes—an effect that is tissue‐specific and temporally distinct from responses observed in the spleen at early timepoints, and not recapitulated by SM‐102 LNPs.

Taken together with previous observations of reduced acute cytokine levels and minimal CD80 upregulation on APCs, these findings suggest that our optimized TRI LNPs provide sufficient short‐term immunomodulatory cues to skew antigen‐specific T cells toward a less inflammatory and potentially more tolerogenic phenotype.

### 2.5 Functional Validation of Optimized TRI LNPs in a Mouse Model of Experimental Autoimmune Encephalomyelitis (EAE)

Having discovered that our optimized TRI LNPs are more effective than SM‐102 LNPs at delivering mRNA without excessively exacerbating inflammation, we sought to validate our TRI LNPs as a therapeutic platform to restore tolerance in inflammatory autoimmune disease. To achieve this, we employed the well‐established mouse model of multiple sclerosis, also known as the EAE mouse model. We evaluated mice treated with TRI LNPs encapsulating Fluc mRNA (TRI Fluc), SM‐102 LNPs encapsulating therapeutic MOG_35‐55_ mRNA (SM‐102 MOG) or TRI LNPs encapsulating therapeutic MOG_35‐55_ mRNA (TRI MOG) via i.m. administration on days 7 and 10 post‐EAE induction. We evaluated the mice for 14 days based on their clinical scores and weights (Figures 6a and S15). Importantly, all experiments were performed in a double blinded scheme to ensure unbiased data collection.

**FIGURE 6:**
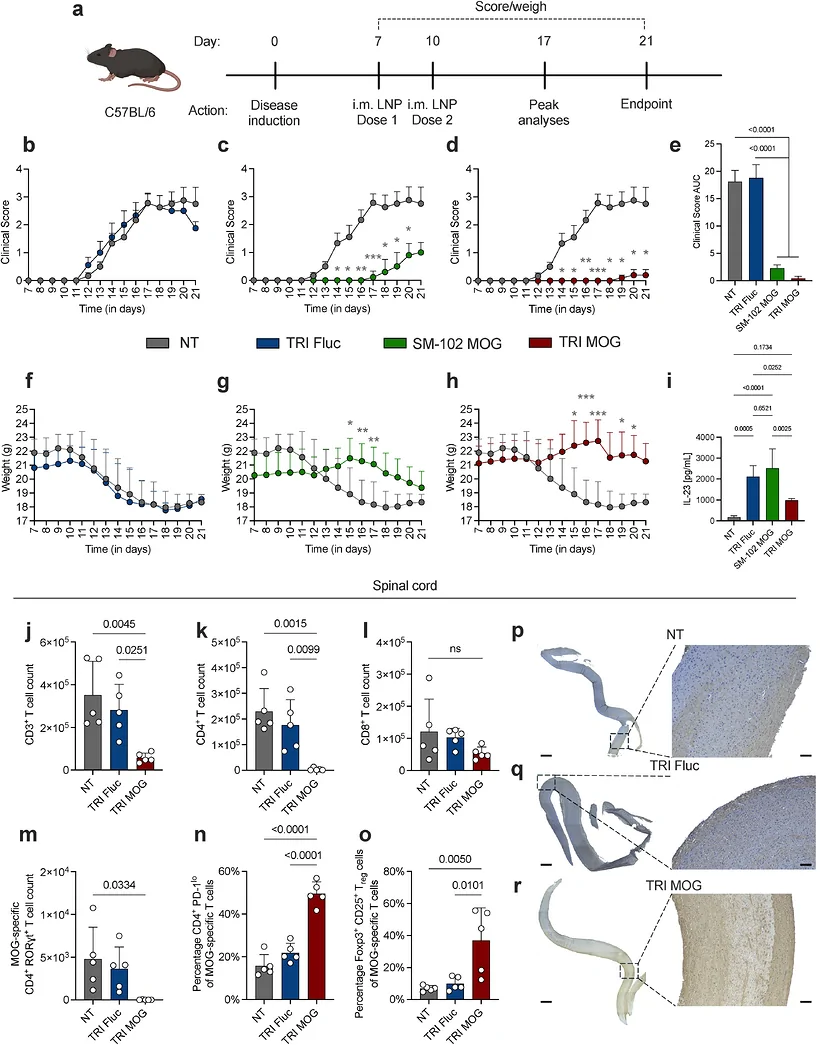
TRI LNPs prevent disease onset and restore tolerance in an experimental autoimmune encephalomyelitis (EAE) mouse model. (a) Experimental design and timeline of LNP treatment in an EAE mouse model. On day 0, an emulsion of complete Freund's adjuvant (CFA) and MOG peptide was subcutaneously administered in C57BL/6 mice, followed by Pertussis toxin i.p. On day 1, Pertussin toxin i.p. was repeated. On day 7 and 10, a 5 µg dose of either TRI LNPs encapsulating Fluc mRNA or TRI LNPs encapsulating MOG_35‐55_ mRNA was administered i.m. Mice that received no LNP treatment (NT) served as a control group. Mice were monitored for clinical scores and weights from day 7 to day 21. Mice were sacrificed on day 17 for peak analyses and on day 21 as the endpoint. (b‐d) EAE clinical disease scores comparing NT vs. (b) TRI Fluc, (c) SM‐102 MOG, and (d) TRI MOG. (e) Corresponding area under curve (AUC) analysis for all groups. (f–h) Body weights of mice in (b–d). Data represent mean + SD for *n* = 10 biological replicates. Statistical differences were determined by two‐sided, one‐way ANOVA with post hoc Tukey's correction. *p < 0.05, **p < 0.01, ***p < 0.001. (i) IL‐23 cytokine levels at peak disease (d17). Data represent mean + SD for *n* = 5 biological replicates. Statistical differences were determined by two‐sided, one‐way ANOVA with post hoc Tukey's correction. (j–o) Quantification via flow cytometry of bulk and antigen‐specific T cell infiltration into mouse spinal cord tissue. Specifically, bulk number of (j) total CD3^+^ T cells, (k) CD4^+^ T cells, and (l) CD8^+^ T cells in spinal cord tissue. (m) Number of antigen‐specific RORɣt^+^ CD4^+^ T cells in spinal cord tissue. Percentage of antigen‐specific (n) PD‐1^lo^ and (o) Foxp3^+^ CD25^+^ T_reg_ cells in spinal cord tissue. Data represent mean + SD for *n* = 5 biological replicates. Statistical differences were determined by two‐sided, one‐way ANOVA with post hoc Tukey's correction. (p–r) Representative brightfield microscopy images of longitudinal histological sections of mouse spinal cord tissue in (p) NT group, (q) TRI Fluc group, and (r) TRI MOG group, with brown regions indicating the presence of myelin. Scale bar in full spinal cord image: 1000 µm. Scale bar in segment of spinal cord image: 50 µm. Data represent *n* = 5 biological replicates.

Mice that received NT or TRI Fluc LNPs exhibited a steady increase in clinical scores until peak disease at day 17. In contrast, mice treated with both SM‐102 MOG and TRI MOG LNPs were strongly protected from paralysis and showed significantly reduced clinical scores from day 14 to 20 (Figure 6b–d). Interestingly, mice treated with SM‐102 MOG LNPs demonstrated a gradual increase in clinical scores from day 17 onward, ending with a mean score of 1 by day 21 (Figures 6c and S16a,c). Mice treated with TRI MOG LNPs demonstrated more durable protection, ending below a mean score of 0.5 by day 21 (Figures 6d and S16b,c). This difference was also evident in the area under the curve analysis (Figure 6e). Additionally, mice in the NT and TRI Fluc groups experienced significant weight loss compared to both SM‐102 and TRI MOG groups over the 14‐day observation period due to their paralysis (Figures 6f–h and S16d–f). Mice treated with SM‐102 MOG LNPs demonstrated significantly preserved body weights from day 15 to 17, with a gradual reduction in body weight from day 18 onward (Figures 6g and S16d,f). However, mice in the TRI MOG group maintained significantly greater body weights compared to NT and TRI Fluc groups until day 20 (Figures 6h and S16e,f).

Notably, there was no significant difference between the clinical scores and body weights of mice treated with SM‐102 MOG and TRI MOG LNPs, indicating that treatment with SM‐102 LNPs may also lead to some level of therapeutic effect. However, cytokine analysis at peak disease indicated that only TRI MOG LNPs were able to prevent a surge in inflammatory cytokine levels in the long‐term. Despite encapsulating therapeutic MOG_35‐55_ mRNA, SM‐102 MOG LNPs significantly elevated levels of IL‐23, IL‐27, and IFN‐β compared to NT (Figures 6i and S16g,h). Similarly, despite containing steroid, TRI Fluc LNPs also significantly elevated cytokine levels compared to NT. However, TRI MOG LNPs did not significantly elevate IL‐23 levels compared to NT and led to significantly lower IL‐23 levels compared to both TRI Fluc and SM‐102 MOG LNP groups (Figure 6i). Although the statistical significance varied, this general trend held true for IL‐27 and IFN‐β levels after TRI MOG treatment as well. Together, our data suggests that a combination of steroid LNP and therapeutic mRNA may be optimal for long‐term reduction in inflammation as well as improved clinical outcomes in EAE.

EAE pathology is characterized by increased T cell infiltration—particularly Th1 and Th17 subsets—into the spinal cord, where they actively mediate the destruction of myelin tissue [35]. To understand the cellular mechanisms of tolerance at play, we harvested spinal cord tissue at peak disease (day 17) and performed flow cytometry (Figure S17). Excitingly, TRI MOG treatment significantly reduced the total number of CD3^+^ T cells in the spinal cord, including reductions in both CD4^+^ and CD8^+^ T cell populations compared to NT and TRI Fluc groups (Figure 6j–l). We further analyzed the MOG‐specific T cell population and found that TRI MOG treatment significantly reduced the number of RORɣt^+^ CD4^+^ T cells, indicating a reduction in Th17 cells, which are key drivers of EAE pathology (Figure 6m) [35]. Moreover, TRI MOG LNPs also reduced the number of antigen‐specific Tbet^+^ RORɣt^+^ CD4^+^ T cells compared to control groups, suggesting a decrease in pathogenic T cells in the Th1‐Th17 transition state (Figure S18a).

We also examined PD‐1 expression within the MOG‐specific T cell population. While previous studies have linked PD‐1/PD‐L1 engagement in conjunction with TCR/major histocompatibility complex (TCR/MHC) signaling to the induction of tolerance, the specific levels of PD‐1 expression required to drive this effect remain unclear [36, 37, 38]. We observed that TRI MOG treatment significantly increased the number of PD‐1^lo^ MOG‐specific T cells compared to control groups, suggesting an association with a tolerogenic state (Figure 6n). Conversely, we observed a significant decrease in PD‐1^hi^ MOG‐specific T cells in the TRI MOG group compared to controls (Figure S18b). These findings suggest that while PD‐1 signaling is important for tolerance, lower levels of PD‐1 expression may be sufficient to support this response, whereas high PD‐1 expression may instead reflect a state of T cell exhaustion rather than tolerance [37].

Importantly, TRI MOG LNP treatment also led to a significant increase in MOG‐specific Foxp3^+^ CD25^+^ T_reg_ cells compared to both control groups (Figure 6o). This indicates that among the antigen‐specific T cells present in the spinal cord, a substantial proportion were T_reg_ cells, which likely contributed to protection of myelin tissue. This protective effect was further confirmed by spinal cord histology. While the NT and TRI Fluc groups exhibited clear signs of myelin destruction, the TRI MOG group demonstrated preservation of myelin integrity (Figure 6p–r). Together, these data validate the ability of i.m. administered TRI LNPs encapsulating therapeutic antigen‐encoding mRNA to induce tolerogenic antigen‐specific T cell phenotypes and prevent onset of disease in a mouse model of EAE.

## 3 Discussion

mRNA LNPs are an extremely versatile delivery platform, owing both to the ability of mRNA to encode virtually any antigen, and to the capacity of LNPs to enable efficient intracellular delivery [3]. Together, they offer a robust and flexible strategy for antigen‐specific immune modulation across various immunological settings, including infectious disease, cancer, and beyond. However, the inherent adjuvanticity of LNPs can trigger downstream inflammation, limiting their safety in conditions where pre‐existing inflammation may be exacerbated [11, 13, 15, 16]. As a result, the application of mRNA LNPs in areas such as inflammatory and autoimmune disease remains severely underexplored [11, 13, 15, 16].

Several approaches have been proposed to mitigate LNP‐induced inflammation, but many require additional immunosuppressive treatments or substantial changes to the chemical structure of ionizable lipids, leading to entirely new LNP formulations with unpredictable efficacy [13]. In contrast, our work introduces a modular solution: incorporating anti‐inflammatory steroids, which are structurally similar to cholesterol, directly into the LNP formulation. These steroids are already FDA‐approved for use in autoimmune diseases, but their systemic toxicity and lack of tissue specificity have limited their broader therapeutic application [39]. By using minimal amounts of steroid as supportive components within the LNP rather than as primary therapeutics, we reduce inflammation associated with LNP delivery while increasing the tolerogenic potential of the mRNA cargo [40]. Moreover, by systematically establishing design rules for LNPs with varying ionizable lipid identities, we aim to create a generalizable platform that can be readily applied to existing LNP formulations while maintaining their intrinsic properties and performance.

In this study, we initially explored six different steroid constructs but only thoroughly characterized and functionally validated TRI incorporation into gold‐standard LNPs. Future work could extend optimization to other steroids, such as MPL and PL, that demonstrated synergy with our LNP platform. Additionally, investigating whether our design principles can be extrapolated to natural sterols such as phytosterols or endogenous corticosteroids could be a valuable next step in expanding our platform as well [41, 42]. Thus, our findings lay the groundwork for establishing steroid incorporation and optimization as a materials design principle for guiding immunomodulatory LNP design.

In summary, our platform established a broadly applicable materials design strategy that overcomes the challenge of LNP adjuvanticity while preserving delivery efficacy, ultimately improving the safety of mRNA LNPs and expanding their potential into inflammatory and autoimmune disease.

## 4 Materials and Methods

### 4.1 LNP Formulation and Characterization

An organic phase containing the ionizable lipid (SM‐102, ALC‐0315, or Dlin‐MC3‐DMA) (Cayman Chemicals), helper lipid (DSPC) (Avanti Research), cholesterol (Millipore Sigma), and PEG‐lipid (DMG‐PEG2K or ALC‐0159) (Avanti Research) was prepared in ethanol at designated molar ratios [12, 27]. This phase was combined with an aqueous phase containing N1‐Methylpseudouridine (m_1_Ψ)‐modified mRNA (FLuc, OVA, or myelin oligodendrocyte glycoprotein (MOG_35‐55_)) using microfluidic mixing to formulate LNPs [20]. LNPs were dialyzed against 1x PBS for 2 h followed by sterile filtration via 0.22 µm filters (Genesee Scientific, Cat. No. 25‐243). For animal experiments, LNPs were concentrated using 2 mL Amicon Ultra Centrifugal Filters with a 50 kDa cutoff (Millipore Sigma, Cat. No. UFC205024) by spinning at 800 × *g* at 4°C. LNPs were concentrated until a concentration of 5 µg mRNA/100 µL (50 ng/µL) was obtained and were kept sterile throughout the process. LNPs were then characterized by measuring size and polydispersity index (PDI) values via dynamic light scattering (Wyatt Technologies) and mRNA encapsulation efficiency via Quant‐iT Ribogreen assay (ThermoFisher) as previously described [43].

### 4.2 Cell Culture

RAW 264.7 and RAW‐blue cells were cultured in DMEM medium (Gibco) containing 10% fetal bovine serum (FBS) and 1% penicillin/streptomycin (P/S). For colorimetric assays, media was switched to phenol red‐free DMEM with 10% FBS and 1% P/S. DC2.4 cells were cultured in RPMI medium (Gibco) containing 10% FBS and 1% P/S. For inducing inflammation, cells were pre‐treated with 0.5 ng/µL LPS derived from E.coli O111:B4 (Millipore Sigma, Cat. No. 437627) in complete medium for 1 h before LNP treatment.

### 4.3 In Vitro mRNA Delivery, ATP Metabolism, and NF‐κB Activity Measurements

RAW‐Blue cells were seeded onto a 96‐well plate at a density of 100,000 cells/well overnight. Cells were then pre‐treated with LPS derived from E.coli O111:B4 (Millipore Sigma) for 1 h, followed by treatment with LNPs encapsulating FLuc mRNA at varying doses for 24 h. Luciferase protein expression was evaluated by using a Luciferase Reporter 1000 Assay System (Promega). Relative cellular ATP metabolism was evaluated by using a CellTiter‐Glo Luminescent Assay System (Promega). NF‐κB activity was evaluated by measuring secreted alkaline phosphatase (SEAP) levels in supernatant using a QUANTI‐Blue Assay (InvivoGen) according to the manufacturer's protocol. All measurements were performed using an Infinite M Plex plate reader (Tecan).

### 4.4 LNP Uptake Studies via Fluorescence Microscopy

RAW 264.7 cells were seeded into 35 mm glass‐bottom dishes (Fisher Scientific) overnight. Cells were then pre‐treated with LPS derived from E.coli O111:B4 (Millipore Sigma) for 1 h, followed by treatment with LNPs labeled with lipophilic Vybrant DiI dye at a 0.5% *v*/*v* ratio (ThermoFisher) at a dose of 100 ng mRNA/100K cells for 4 h. Next, cells were stained with Hoechst 33 342 (5 µg/mL) (ThermoFisher) for 10 min at 37°C, washed with 1x PBS twice, and then stained with LysoTracker Deep Red (100 nM) (ThermoFisher) for 30 min at 37°C. Finally, cells were washed with 1X PBS and incubated in phenol‐red free DMEM for imaging. Images were taken immediately using a confocal laser scanning microscope (Leica Stellaris 5). Images were acquired in a single plane of focus and were acquired based on regions that contained a minimum of 10 cells in the field of view. *N* = 3 experiments were performed, and *n* = 3 fields of view were imaged per treatment group per experiment. In each field of view, *n* = 5 cells were randomly chosen and analyzed to calculate percent decolocalized LNP signal. Percent decolocalized signal was calculated as follows: [MFI Pixel (LNP channel)—MFI Pixel (Colocalized Channel)]/[MFI Pixel (LNP channel)].

### 4.5 RT‐qPCR and ELISA on Inflamed RAW 264.7 Cells

RAW 264.7 cells were pre‐treated with or without LPS (0.5 ng/µL) for 1 h and then treated with an LNP library of DLin‐MC3‐DMA (MC3), C12‐200, 4A3‐SC8, SM‐102, or TRI LNPs at 100 ng mRNA/100,000 cells. Cells and supernatant were harvested 6 h post‐treatment for analysis. Cells were washed with cold 1x PBS, lysed, and total RNA was extracted using RNeasy Mini Kit (Qiagen) as per the manufacturer's instructions. The concentration of eluted RNA was quantified using Nanodrop (Thermo Scientific) and aliquoted and stored at −80°C for later use. A Luna Universal One‐Step RT‐qPCR kit (New England Biolabs, Cat. No. E3005) was used to perform RT‐qPCR as per the manufacturer's instructions. 100 ng RNA was used as template RNA in the reaction. RT‐qPCR was run on a QuantStudio 5 Real‐Time PCR System (Applied Biosystems, Fisher Scientific) as previously described. Data analysis was performed using the ΔΔ*C*_t_ method [44]. A mouse IL‐6 ELISA kit (Thermo Fisher) was used to evaluate IL‐6 levels in supernatant as per the manufacturer's instructions.

### 4.6 Animals

All animal protocols were approved by the Institutional Animal Care and Use Committee (IACUC) of the University of Pennsylvania (806540), and animal procedures were performed in accordance with the Guidelines for Care and Use of Laboratory Animals at the University of Pennsylvania. Female C57BL/6 mice (8–10 weeks of age, 22–24 g body weight) were purchased from Jackson Laboratory. Both male and female OT‐II mice on a C57BL/6 background were purchased from Jackson Laboratory and bred in‐house. All animals were housed in a specific‐pathogen‐free animal facility at ambient temperature (22°C ± 2°C), air humidity 40%–70% and 12 h dark/light cycles.

### 4.7 Endotoxemia Mouse Model

At 0 h, mice were injected intraperitoneally (i.p.) with 0.1 mg/kg LPS derived from E.coli O111:B4 (Millipore Sigma) to induce systemic inflammation. At 4 h post‐LPS, mice were injected intramuscularly (i.m.) with LNPs labeled with lipophilic Vybrant DiD dye (ThermoFisher) at 0.5% *v*/*v* ratio and encapsulating FLuc mRNA at a dose of 5 µg mRNA/mouse or 1x PBS. Note: the treatment dose was split across both hind limbs of the mice. At 4 h post‐treatment, mice were euthanized, and major organs (heart, lungs, liver, kidneys, and spleen) and lymph nodes (inguinal and iliac) were collected. mRNA delivery and LNP accumulation were quantified using an in vivo imaging system (IVIS). For cell‐specific uptake studies, LNPs were dyed with lipophilic DiD dye at a 0.5% *v*/*v* ratio and were injected intramuscularly as described. At 4h‐post treatment, mice were euthanized, and the inguinal lymph nodes and iliac lymph nodes were harvested, mechanically digested through a 70 µm cell‐strainer (Falcon), treated with ACK Lysis buffer (Quality Biological) and quenched with serum‐containing DMEM to obtain single cell suspensions. Cells were then washed with 1x PBS, counted, surface‐stained and analyzed for LNP uptake via flow cytometry.

### 4.8 Serum Cytokine Measurements

Blood from mice was collected into serum collection tubes (BD) using retroorbital bleeding and allowed to clot for 2 h at 25°C. Tubes were centrifuged at 2000 × *g* for 20 mins to isolate serum. Serum was then carefully collected and stored at −20°C before analysis. All serum was analyzed using a multiplexed flow cytometry‐based LEGENDplex assay (Biolegend) on an iQue3 Screener (Sartorius) as per the manufacturer's instructions.

### 4.9 OT‐II Adoptive Transfer Mouse Model

On day 0, OT‐II CD4^+^ T cells were isolated from spleens of donor OT‐II TCR transgenic mice using a negative selection‐based CD4^+^ T cell isolation kit (Biolegend). OT‐II cells were then assessed for viability, counted using a Countess Cell Counter (ThermoFisher) and were stained with CellTrace Violet (ThermoFisher) following the manufacturer's protocol. After staining, cells were quenched with serum‐containing DMEM, centrifuged and resuspended in 1x PBS. Cells were then injected intravenously (i.v.) into recipient female C57BL/6 mice, with each mouse receiving 1 × 10^6^ cells. Mice were injected i.p. with 0.1 mg/kg of LPS (Millipore Sigma) immediately after to induce systemic inflammation. On day 1, LNPs encapsulating OVA‐encoding mRNA were administered i.m. at a dose of 5 µg mRNA/mouse, followed by 0.1 mg/kg LPS i.p. Note: the treatment dose was split across both hind limbs of the mice as done previously. On day 3, recipient mice were euthanized and spleens, inguinal lymph nodes, iliac lymph nodes, and axillary lymph nodes were harvested and processed into single cell suspensions for restimulation and flow cytometric analysis.

### 4.10 Ex Vivo Restimulation

Single cell suspensions from mouse spleen and lymph nodes were counted. 1–3 million cells were restimulated in a 48‐well plate with OVA_323‐339_ peptide (GenScript) and anti‐CD28 (Tonbo biosciences) each at 2.5 µg/mL for 6 h. At 2 h post‐restimulation, 2 µL of monensin (BD) was added per sample. Unstimulated wells were included as controls to determine background levels of nonspecific activation. Cells were then washed with 1x PBS and stained for flow cytometriy.

### 4.11 EAE Model

All experiments were double blinded to ensure unbiased data collection and interpretation. EAE was induced in female C57BL/6 mice (8–10 weeks) according to EAE induction instructions from Hooke laboratory. 110 ng of Pertussis toxin (PTX) in 1x PBS was administered i.p. on day 0 and 1 post‐EAE induction. Mice were injected with 1x PBS or 5 µg mRNA LNPs i.m. on day 7 and 10 post‐EAE induction. Note: the treatment dose was split across both hind limbs of the mice as done previously. Mice were monitored daily from day 7 to 21 and evaluated based on clinical score and body weight. Clinical scoring guidelines are outlined in Figure S13. In a separate experiment, mice were euthanized on day 17, which is peak disease, and perfused with 1x PBS to clear residual blood from their system. Spinal cords were then harvested and either fixed for histology or analyzed via flow cytometry. For flow cytometry, spinal cords were processed into single cell suspensions using a Neural Tissue Dissociation Kit (Milltenyi Biotech) and a gentleMACS Octo dissociator (Milltenyi Biotech) according to the manufacturer's protocol.

### 4.12 Flow Cytometry

For in vitro uptake studies, RAW 264.7 cells and DC2.4 cells were stained with 1:1000 Zombie NIR Live/Dead fixable dye (Biolegend) and analyzed using a Luminex Guava easycyte benchtop flow cytometer (Luminex) equipped with Incyte software (Guava). For in vivo cell‐specific LNP uptake, cells were stained with 1:200 CD16/CD32 Fc Block (Biolegend) and 1:1000 Zombie UV live/dead fixable dye (Biolegend) at 4°C for 20 min. Cells were then washed and stained with surface antibodies at 4°C for 30 min. For phenotypic analysis in the OT‐II adoptive transfer model, cells were stained with Fc Block and Zombie NIR Live/Dead fixable dye and then fixed and permeabilized using the FoxP3 Transcription Factor Kit (eBioscience) according to the manufacturer's protocol. Cells were then stained with surface and intracellular antibodies simultaneously at 4°C for 45 min. For MOG‐specific T cell analysis, cells were stained with Fc Block, Zombie NIR Live/Dead fixable dye and then stained with 1:100 MOG‐tetramer‐PE (NIH Tetramer facility) at 37°C for 1 h. Cells were then fixed and permeabilized as outlined above and stained with surface and intracellular antibodies. All in vivo samples were stored overnight in 1x PBS and analyzed using spectral flow cytometry on a Symphony A5 SE flow cytometer (BD).

### 4.13 Histology

Spinal cords of mice were collected to assess changes in myelin at peak disease (day 17) in the EAE model. Mice were perfused with 1x PBS and spinal cords were harvested and placed in 10% neutral‐buffered formalin (Millipore Sigma) for 24 h at 4°C to fix. Samples were then dehydrated in ethanol and embedded in paraffin wax. A 4 µm longitudinal section was taken along the entire length of the spinal cord and stained using a primary anti‐myelin basic protein antibody (Millipore Sigma) at a 1:100 dilution, followed by horseradish peroxidase chromogenic detection (Vector labs). Brightfield microscopy images were taken using an EVOS M7000 Imager (ThermoFisher) with 4x and 10x objectives.

### 4.14 Statistical Analysis

All plots and statistics were visualized and calculated using GraphPad Prism. FlowJo was used to analyze flow cytometry data. ImageJ2 was used to analyze confocal laser scanning microscopy images. For in vitro luciferase mRNA delivery studies, all samples were normalized to samples with no LNP treatment, as noted in the figure captions. Absolute cell counts in the OT‐II adoptive transfer studies and EAE studies were determined by including Precision Count Beads (Biolegend) in our flow samples and following the manufacturer's instructions for count calculation. For EAE studies, all participants were double blinded to ensure unbiased data collection and interpretation.

All data is presented as mean + standard deviation or mean ± standard deviation (SD) with the exact number of replicates reported in the figure legends. All samples had at least *n* = 3 biological replicates, and no statistical methods were used to predetermine sample size. For multiple comparisons, two‐sided, one‐way ANOVA tests were followed by post hoc Tukey's correction. Statistical tests and post hoc correction choices are reported in the figure legends.

## Author Contributions

A.S.T., J.R.M., and M.J.M. conceived the project and designed the experiments. The experiments were performed by A.S.T., B.E.N., J.B.P., J.M.R, L.C., H.C.S., H.C.G., H.M.Y., M.J.Y.A., M.K., Q.S., J.W., E.B., Y.Z., A.G.H., and K.L.S. with guidance from D.W. and interpreted by all authors. A.S.T., J.R.M. and M.J.M. wrote the article, and A.S.T. prepared the figures in Biorender and Prism. All authors edited the article and figures and approved the final version for submission.

## Conflicts of Interest

The authors declare no conflicts of interest.

## Supporting information




**Supporting File**: adma74301‐sup‐0001‐SuppMat.docx.

## Data Availability

All data supporting the results in this study are available within the article and the Supporting Information. The raw data generated during the study are available for research purposes from the corresponding authors on reasonable request.
