# Supplementary material for: Ionizable Lipid‐Dependent Optimization of Steroid Lipid Nanoparticles With Tunable Immunomodulatory Properties
Source: Adv Mater. 2026 Aug 6;38(56):e74301. doi: 10.1002/adma.74301 (PMC13648879; doi:10.1002/adma.74301)
Supplement: Supplementary file 1 [file ADMA-38-e74301-s001.docx]

**Ionizable lipid-dependent optimization of steroid lipid nanoparticles with tunable immunomodulatory properties**

Ajay S. Thatte^1^, Benjamin E. Nachod^1^, Julia Baena^1^, Jenna Muscat-Rivera^2,3^, Lesley Chaboub^2,3^, Hannah C. Safford^1^, Hannah C. Geisler^1^, Hannah M. Yamagata^1^, Melgious J. Y. Ang^1^, Michael Kegel^2,3^, Alexandre Poirier^1^, Elaine R. Tong^1^, Zhe Zhong^1^, Qiangqiang Shi^1^, Jinjin Wang^1^, Elisa Battistini^1^, Ye Zeng^1^, Alex G. Hamilton^1^, Kelsey L. Swingle^1^, Drew Weissman^2,3^, Jilian R. Melamed^2,3^, Michael J. Mitchell^1,3,4,5,6,7*^

**AFFILIATIONS**

^1^Department of Bioengineering, University of Pennsylvania, Philadelphia, PA 19104 USA.

^2^Department of Medicine, University of Pennsylvania, Philadelphia, PA 19104 USA.

^3^Penn Institute for RNA Innovation, Perelman School of Medicine, University of Pennsylvania, Philadelphia, PA 19104 USA.

^4^Institute for Immunology, Perelman School of Medicine, University of Pennsylvania, Philadelphia, PA 19104 USA.

^5^Cardiovascular Institute, Perelman School of Medicine, University of Pennsylvania, Philadelphia, PA 19104 USA.

^6^Institute for Regenerative Medicine, Perelman School of Medicine, University of Pennsylvania, Philadelphia, PA 19104 USA.

^7^Abramson Cancer Center, Perelman School of Medicine, University of Pennsylvania, Philadelphia, PA 19104 USA.

**CORRESPONDING AUTHORS**

| *Michael J. Mitchell, Ph.D.  Associate Professor  Department of Bioengineering  University of Pennsylvania  25 N 38^th^ St, Suite 1014  Philadelphia, PA 19104, USA  [mjmitch@seas.upenn.edu](mailto:mjmitch@seas.upenn.edu) | *Jilian R. Melamed, Ph.D.  Research Assistant Professor  Perelman School of Medicine  University of Pennsylvania  25 N 38^th^ St, Room 4072  Philadelphia, PA 19104, USA  [jilian.melamed@pennmedicine.upenn.edu](mailto:jilian.melamed@pennmedicine.upenn.edu) |
| --- | --- |

**TABLE OF CONTENTS**

**Supplementary Figure S1** | NF-κB activation upon free steroid or steroid-LNP treatment *in vitro*.

**Supplementary Figure S2** | Steroid structures with annotated carbons.

**Supplementary Figure S3** | DiI-dyed LNP uptake in RAW 264.7 and DC2.4 cells *in vitro*.

**Supplementary Figure S4** | Confocal fluorescence microscopy visualizing LNP uptake in inflamed RAW 264.7 cells.

**Supplementary Figure S5** | LNP-induced inflammation in RAW 264.7 cells *in vitro*.

**Supplementary Figure S6** | Stability measurements for Base (SM-102) LNPs and TRI LNPs.

**Supplementary Figure S7** | mRNA delivery and NF-κB activation of industry-standard LNPs with varying TRI substitution *in vitro*.

**Supplementary Figure S8** | LNP accumulation after i.m. administration in endotoxemia mouse model.

**Supplementary Figure S9** | Fluc mRNA delivery to major organs after i.m. administration in endotoxemia mouse model.

**Supplementary Figure S10** | Representative gating strategy for peripheral lymph nodes in endotoxemia mouse model.

**Supplementary Figure S11** | DiD-dyed LNP uptake by lymphocytes upon i.m. administration in endotoxemia mouse model.

**Supplementary Figure S12** | Individual cytokine levels upon i.m. administration of LNPs in healthy mice.

**Supplementary Figure S13** | Individual cytokine levels upon i.m. administration of LNPs in endotoxemia mouse model.

**Supplementary Figure S14** | Representative gating strategy for spleen and peripheral lymph nodes in OT-II adoptive transfer model.

**Supplementary Figure S15** | EAE clinical scoring guidelines.

**Supplementary Figure S16** | Further comparison of EAE clinical scores, body weights and cytokines.

**Supplementary Figure S17** | Representative gating strategy for immune cells in spinal cord in EAE model.

**Supplementary Figure S18** | Other immune phenotyping of T cells present in spinal cord in EAE model.

**Supplementary Table S1** | Size and polydispersity of SM-102 LNPs with 50% substitution of cholesterol for each steroid.

**Supplementary Table S2** | P-values obtained from comparison between relative ATP abundance in RAW 264.7 cells upon steroid LNP treatment. Statistical differences were determined by two-sided, one-way ANOVA with *post hoc* Tukey’s comparison.

**Supplementary Table S3** | Size and polydispersity of SM-102 LNPs with 0%, 30%, 50% and 80% TRI substitution for cholesterol.

**Supplementary Table S4** | P-values obtained from size comparison between Base (SM-102) LNPs and 50% TRI-substituted LNPs over 28 days. Statistical differences were determined by two-sided, two-way ANOVA with *post hoc* Tukey’s comparison.

**Supplementary Table S5** | P-values obtained from PDI comparison between Base (SM-102) LNPs and 50% TRI-substituted LNPs over 28 days. Statistical differences were determined by two-sided, two-way ANOVA with *post hoc* Tukey’s comparison.

**Supplementary Table S6** | P-values obtained from mRNA encapsulation efficiency comparison between Base (SM-102) LNPs and 50% TRI-substituted LNPs over 28 days. Statistical differences were determined by two-sided, two-way ANOVA with *post hoc* Tukey’s comparison.

**Supplementary Table S7** | Size and polydispersity of ALC-0315 LNPs with 0%, 30%, 50% and 80% TRI substitution for cholesterol.

**Supplementary Table S8** | Size and polydispersity of DLin-MC3-DMA LNPs with 0%, 30%, 50% and 80% TRI substitution for cholesterol.

**SUPPLEMENTARY FIGURES**

**Figure S1: NF-κB activation upon free steroid or steroid-LNP treatment *in vitro*.** RAW-Blue cells pre-treated with LPS were treated with free dexamethasone (Dex), SM-102 LNP, or SM-102 LNP containing 10% or 50% substitution of cholesterol for Dex. Levels of NF-κB activation at varying steroid doses were quantified via SEAP reporter expression in the supernatant. Data was normalized to cells with no treatment. Data represent mean ± SD for n=3 biological replicates. Statistical differences were determined by two-sided, one-way ANOVA with *post hoc* Tukey’s correction.

**Figure S2: Steroid structures with annotated carbons.** Numbered carbons for (a) dexamethasone, (b) hydrocortisone, (c) methylprednisolone, (d) prednisolone, (e) prednisone and (f) triamcinolone.

**Figure S3: DiI-dyed LNP uptake in RAW 264.7 and DC2.4 cells *in vitro*.** Quantification of DiI^+^ RAW 264.7 cells (a) 1h post-treatment and (b) 4h post-treatment via flow cytometry. Quantification of DC2.4 cells (a) 1h post-treatment and (b) 4h post-treatment via flow cytometry. Data represent mean + SD for n=4 biological replicates. Statistical differences were determined by two-sided, one-way ANOVA with *post hoc* Tukey’s correction.

**Figure S4: Confocal fluorescence microscopy visualizing LNP uptake in inflamed RAW 264.7 cells.** RAW 264.7 cells were pre-treated with LPS for 1h and were then treated with DiI-labeled Base (0%), 30%, 50% and 80% TRI substituted LNPs for 4h at a dose of 100 ng mRNA/100K cells. Images taken at 25x magnification. Scale bar: 50 μm.

**Figure S5: LNP-induced inflammation in RAW 264.7 cells *in vitro*.** RAW 264.7 cells were pre-treated with or without LPS (1 ng/mL) for 1h and then treated with an LNP library of DLin-MC3-DMA (MC3), C12-200, 4A3-SC8, SM-102 or TRI LNPs at 100 ng mRNA/100K cells. Cells and supernatant were harvested 6h post-treatment to analyze (a) fold change in CCL2 gene expression and (b) IL-6 levels. Data represent mean + SD for n=3 biological replicates. Statistical differences were determined by two-sided, two-way ANOVA with *post hoc* Tukey’s correction.

**Figure S6: Stability measurements for Base (SM-102) LNPs and TRI LNPs**. (a) LNP size (in nm), (b) polydispersity indices (PDIs), and (c) mRNA encapsulation efficiency for both Base and 50% TRI-substituted LNPs across 28 days with storage at 4°C. Data represent mean + SD for n=3-4 replicates. Statistical differences were determined by two-sided, two-way ANOVA with *post hoc* Tukey’s correction. Pairwise comparisons displayed on graphs. Full list of multiple comparisons and respective p-values listed in Supplementary Tables S2-4.

**Figure S7: mRNA delivery and NF-κB activation of industry-standard LNPs with varying TRI substitution *in vitro*.** (a) Relative luminescence and (b) corresponding NF-κB activation in RAW-Blue cells pre-treated with LPS upon mRNA delivery via Dlin-MC3-DMA (MC3) LNP. Data represent n=3 biological replicates. (c) Relative luminescence and (d) corresponding NF-κB activation in RAW-Blue cells pre-treated with LPS upon mRNA delivery via ALC-0315 LNP. Data represent mean + SD for n=3-6 biological replicates. Statistical differences were determined by two-sided, two-way ANOVA with *post hoc* Tukey’s correction.

**Figure S8:** **LNP accumulation after i.m. administration in endotoxemia mouse model.** (a) IVIS image visualizing DiD-dyed LNP accumulation in major organs and lymph nodes. (b-f) Quantification of DiD fluorescence in (b) lungs, (c) liver, (d) spleen, (e) inguinal lymph nodes and (f) iliac lymph nodes upon i.m. administration. Data represent mean + SD for n=3 biological replicates. Statistical differences were determined by two-sided, one-way ANOVA with *post hoc* Tukey’s correction.

**Figure S9: Fluc mRNA delivery to major organs after i.m. administration in endotoxemia mouse model.** (a-c) Quantification of luminescence in (a) lungs, (b) liver and (c) spleen upon i.m. administration. Data represent mean + SD for n=3 biological replicates. Statistical differences were determined by two-sided, one-way ANOVA with *post hoc* Tukey’s correction.

**Figure S10: Representative gating strategy for peripheral lymph nodes in endotoxemia mouse model.** Quantification of n=4 biological replicates provided in the main text. Statistical differences were determined by two-sided, one-way ANOVA with *post hoc* Tukey’s correction.

**Figure S11: LNP uptake by lymphocytes upon i.m. administration in endotoxemia mouse model.** Quantification of DiD-LNP^+^ cells in various lymphocyte subsets: (a) CD19^+^ B cells, (b) Total CD3^+^ T cells, (c) CD4^+^ T cells, and (d) CD8^+^ T cells. Data represent mean + SD for n=4 biological replicates. Statistical differences were determined by two-sided, one-way ANOVA with *post hoc* Tukey’s correction.

**Figure S12: Individual cytokine levels upon i.m. administration of LNPs in healthy mice.** (a-d) Individual cytokine levels of (a) IFN-β, (b) GM-CSF, (c) IL-1⍺, and (d) IL-10 upon i.m. administration of SM-102 or TRI LNPs in healthy mice at a dose of 5 μg. Data represent mean + SD for n=4 biological replicates. Statistical differences were determined by two-sided, one-way ANOVA with *post hoc* Tukey’s correction.

**Figure S13: Individual cytokine levels upon i.m. administration of LNPs in endotoxemia mouse model.** Quantification of (a) IL-23, (b) IFN-ɣ, (c) TNF, (d) MCP-1, (e) IL-12p70, (f) IL-1β, (g) IL-10, (h) IL-27, (i) IL-17a. Data represent mean ± SD for n=3-4 biological replicates. Statistical differences were determined by two-sided, one-way ANOVA with *post hoc* Tukey’s correction.

**Figure S14: Representative gating strategy for spleen and peripheral lymph nodes in OT-II adoptive transfer model.** Quantification of n=4 biological replicates provided in the main text. Statistical differences were determined by two-sided, one-way ANOVA with *post hoc* Tukey’s correction.

| **Score** | **Clinical Observation** |
| --- | --- |
| 0.0 | No obvious changes in motor function compared to non-immunized mice  When picked up by base of tail, the tail has tension and is erect. Hind legs are usually spread apart. When the mouse is walking, there is no gait or head titling. |
| 0.5 | Tip of tail is limp.  When picked up by base of tail, the tail has tension except for the tip. Muscle straining is felt in the tail, while the tail continues to move. |
| 1.0 | Limp tail.  When picked up by base of tail, instead of being erect, the whole tail drapes over finger. Hind legs are usually spread apart. No signs of tail movement are observed. |
| 1.5 | Limp tail and hind leg inhibition.  When picked up by base of tail, the whole tail drapes over finger. When the mouse is dropped on a wire rack, at least one hind leg falls through consistently. Walking is very slightly wobbly. |
| 2.0 | Limp tail and weakness of hind legs.  When picked up by base of tail, the legs are not spread apart but held closer together. When the mouse is observed walking, it has a clearly apparent wobbly walk. One foot may have toes dragging, but the other leg has no apparent inhibitions of movement.  -OR-  Mouse appears to be at score 0.0, but there are obvious signs of head tilting when the walk is observed. The balance is poor. |
| 2.5 | Limp tail and dragging of hind legs.  Both hind legs have some movement, but both are dragging at the feet (mouse trips on hind feet).  -OR-  No movement in one leg/completely dragging one leg, but movement in the other leg.  -OR-  EAE severity appears mild when picked up (as score 0.0-1.5), but there is a strong head tilt that causes the mouse to occasionally fall over. |
| 3.0 | Limp tail and complete paralysis of hind legs (most common).  -OR-  Limp tail and almost complete paralysis of hind legs. One or both hind legs are able to paddle, but neither hind leg is able to move forward of the hind hip.  -OR-  Limp tail with paralysis of one front and one hind leg.  -OR-  All of the following:   - Severe head tilting - Walking only along the edges of the cage - Pushing against the cage wall - Spinning when picked up by base of tail |
| 3.5 | Limp tail and complete paralysis of hind legs. In addition to:  Mouse is moving around the cage, but when placed on its side, is unable to right itself. Hind legs are together on one side of body.  -OR-  Mouse is moving around the cage, but the hind quarters are flat like a pancake, giving the appearance of a hump in front quarters of the mouse. |
| 4.0 | Limp tail, complete hind leg and partial front leg paralysis.  Mouse is minimally moving around the cage but appears alert and feeding.  Often euthanasia is recommended after the mouse scores 4.0 for 2 days. However, with daily s.c. fluids, most C57BL/6 mice may recover to 3.5 or 3.0, while SJL mice may fully recover even if they reach score 4.0 at the peak of disease. When the mouse is euthanized because of severe paralysis, a score of 5.0 is entered for that mouse for the rest of the experiment. |
| 4.5 | Complete hind and partial front leg paralysis, no movement around the cage. Mouse is not alert.  Mouse has minimal movement in the front legs. The mouse barely responds to contact.  Euthanasia is recommended. When the mouse is euthanized because of severe paralysis, a score of 5.0 is entered for that mouse for the rest of the experiment. |
| 5.0 | Mouse is spontaneously rolling in the cage (euthanasia is recommended).  -OR-  Mouse is found dead due to paralysis.  -OR-  Mouse is euthanized due to severe paralysis. |

**Figure S15: EAE clinical scoring guidelines.** Guidelines were strictly followed across all groups in all experiments.

**Figure S16: Further comparison of EAE clinical scores, body weights and cytokines.** (a-c) EAE clinical disease scores comparing (a) TRI Fluc vs SM-102 MOG, (b) TRI Fluc vs TRI MOG and (c) SM-102 MOG vs TRI MOG. (d-f) Body weights of mice in (a-c). Data represent mean + SD for n=10 biological replicates. Statistical differences were determined by two-sided, one-way ANOVA with *post hoc* Tukey’s correction. *p<0.05, **p<0.01, ***p<0.001, ****p<0.0001. (g-h) Quantification of (g) IL-27 and (h) IFN-β cytokine levels at peak disease (d17). Data represent mean + SD for n=5 biological replicates. Statistical differences were determined by two-sided, one-way ANOVA with *post hoc* Tukey’s correction.

**Figure S17: Representative gating strategy for immune cells in spinal cord in EAE model.** Quantification of n=5 biological replicates provided in the main text. Statistical differences were determined by two-sided, one-way ANOVA with *post hoc* Tukey’s correction.

**Figure S18: Other immune phenotyping of T cells present in spinal cord in EAE model.** (a) Quantification of the number of MOG-specific T cells expressing both Tbet and RORɣt, potentially indicating a Th1 to Th17 transition phenotype. (b) Quantification of percentage of MOG-specific CD4^+^ T cells expressing high levels of PD-1, potentially indicating an exhausted phenotype. Data represent mean + SD for n=5 biological replicates. Statistical differences were determined by two-sided, one-way ANOVA with *post hoc* Tukey’s correction.

**SUPPLEMENTARY TABLES**

**Table S1:** Size and polydispersity of SM-102 LNPs with 50% substitution of cholesterol for each steroid.

| LNP | Diameter (nm) | Polydispersity Index (PDI) |
| --- | --- | --- |
| SM-102 (no steroid) | 78.7 ± 30.1 | 0.15 ± 0.01 |
| SM-102 + DEX | 89.1 ± 33.9 | 0.14 ± 0.02 |
| SM-102 + P | 83.6 ± 27.4 | 0.11 ± 0.01 |
| SM-102 + PL | 82.1 ± 29.8 | 0.13 ± 0.02 |
| SM-102 + MPL | 81.9 ± 30.3 | 0.14 ± 0.07 |
| SM-102 + TRI | 83.3 ± 28.3 | 0.12 ± 0.01 |
| SM-102 + HYDRO | 76.6 ± 16.2 | 0.05 ± 0.02 |

**Table S2:** P-values obtained from comparison between relative ATP abundance in RAW 264.7 cells upon steroid LNP treatment. Statistical differences were determined by two-sided, one-way ANOVA with *post hoc* Tukey’s comparison.

| Tukey's multiple comparisons test | Adjusted P-Value |
| --- | --- |
| Base vs. DEX | 0.9844 |
| Base vs. P | 0.4708 |
| Base vs. PL | 0.0100 |
| Base vs. MPL | 0.0002 |
| Base vs. TRI | 0.0021 |
| Base vs. HYD | 0.9829 |
| DEX vs. P | 0.8824 |
| DEX vs. PL | 0.0405 |
| DEX vs. MPL | 0.0006 |
| DEX vs. TRI | 0.0084 |
| DEX vs. HYD | >0.9999 |
| P vs. PL | 0.2953 |
| P vs. MPL | 0.0052 |
| P vs. TRI | 0.0735 |
| P vs. HYD | 0.8877 |
| PL vs. MPL | 0.2965 |
| PL vs. TRI | 0.9723 |
| PL vs. HYD | 0.0415 |
| MPL vs. TRI | 0.7609 |
| MPL vs. HYD | 0.0007 |
| TRI vs. HYD | 0.0086 |

**Table S3:** Size and polydispersity of SM-102 LNPs with 0%, 30%, 50% and 80% TRI substitution for cholesterol.

| LNP | Diameter (nm) | Polydispersity Index (PDI) |
| --- | --- | --- |
| 0% | 92.1 ± 32.8 | 0.21 ± 0.02 |
| 30% | 71.9 ± 25.0 | 0.12 ± 0.02 |
| 50% | 83.3 ± 28.3 | 0.12 ± 0.01 |
| 80% | 76.7 ± 15.6 | 0.04 ± 0.01 |

**Table S4:** P-values obtained from size comparison between Base (SM-102) LNPs and 50% TRI-substituted LNPs over 28 days. Statistical differences were determined by two-sided, two-way ANOVA with *post hoc* Tukey’s comparison.

| Tukey’s multiple comparisons test | Adjusted P-value |
| --- | --- |
| 1:Base vs. 1:TRI | >0.9999 |
| 1:Base vs. 2:Base | >0.9999 |
| 1:Base vs. 2:TRI | >0.9999 |
| 1:Base vs. 3:Base | 0.0007 |
| 1:Base vs. 3:TRI | >0.9999 |
| 1:Base vs. 4:Base | 0.0002 |
| 1:Base vs. 4:TRI | 0.0002 |
| 1:Base vs. 7:Base | >0.9999 |
| 1:Base vs. 7:TRI | >0.9999 |
| 1:Base vs. 11:Base | <0.0001 |
| 1:Base vs. 11:TRI | 0.2439 |
| 1:Base vs. 14:Base | <0.0001 |
| 1:Base vs. 14:TRI | <0.0001 |
| 1:Base vs. 28:Base | <0.0001 |
| 1:Base vs. 28:TRI | <0.0001 |
| 1:TRI vs. 2:Base | >0.9999 |
| 1:TRI vs. 2:TRI | >0.9999 |
| 1:TRI vs. 3:Base | 0.0007 |
| 1:TRI vs. 3:TRI | >0.9999 |
| 1:TRI vs. 4:Base | 0.0002 |
| 1:TRI vs. 4:TRI | 0.0002 |
| 1:TRI vs. 7:Base | >0.9999 |
| 1:TRI vs. 7:TRI | >0.9999 |
| 1:TRI vs. 11:Base | <0.0001 |
| 1:TRI vs. 11:TRI | 0.2439 |
| 1:TRI vs. 14:Base | <0.0001 |
| 1:TRI vs. 14:TRI | <0.0001 |
| 1:TRI vs. 28:Base | <0.0001 |
| 1:TRI vs. 28:TRI | <0.0001 |
| 2:Base vs. 2:TRI | >0.9999 |
| 2:Base vs. 3:Base | 0.0007 |
| 2:Base vs. 3:TRI | >0.9999 |
| 2:Base vs. 4:Base | 0.0002 |
| 2:Base vs. 4:TRI | 0.0002 |
| 2:Base vs. 7:Base | >0.9999 |
| 2:Base vs. 7:TRI | >0.9999 |
| 2:Base vs. 11:Base | <0.0001 |
| 2:Base vs. 11:TRI | 0.2439 |
| 2:Base vs. 14:Base | <0.0001 |
| 2:Base vs. 14:TRI | <0.0001 |
| 2:Base vs. 28:Base | <0.0001 |
| 2:Base vs. 28:TRI | <0.0001 |
| 2:TRI vs. 3:Base | 0.0007 |
| 2:TRI vs. 3:TRI | >0.9999 |
| 2:TRI vs. 4:Base | 0.0002 |
| 2:TRI vs. 4:TRI | 0.0002 |
| 2:TRI vs. 7:Base | >0.9999 |
| 2:TRI vs. 7:TRI | >0.9999 |
| 2:TRI vs. 11:Base | <0.0001 |
| 2:TRI vs. 11:TRI | 0.2439 |
| 2:TRI vs. 14:Base | <0.0001 |
| 2:TRI vs. 14:TRI | <0.0001 |
| 2:TRI vs. 28:Base | <0.0001 |
| 2:TRI vs. 28:TRI | <0.0001 |
| 3:Base vs. 3:TRI | 0.0072 |
| 3:Base vs. 4:Base | <0.0001 |
| 3:Base vs. 4:TRI | <0.0001 |
| 3:Base vs. 7:Base | 0.0007 |
| 3:Base vs. 7:TRI | 0.0007 |
| 3:Base vs. 11:Base | <0.0001 |
| 3:Base vs. 11:TRI | <0.0001 |
| 3:Base vs. 14:Base | <0.0001 |
| 3:Base vs. 14:TRI | <0.0001 |
| 3:Base vs. 28:Base | <0.0001 |
| 3:Base vs. 28:TRI | <0.0001 |
| 3:TRI vs. 4:Base | <0.0001 |
| 3:TRI vs. 4:TRI | <0.0001 |
| 3:TRI vs. 7:Base | >0.9999 |
| 3:TRI vs. 7:TRI | >0.9999 |
| 3:TRI vs. 11:Base | <0.0001 |
| 3:TRI vs. 11:TRI | 0.0409 |
| 3:TRI vs. 14:Base | <0.0001 |
| 3:TRI vs. 14:TRI | <0.0001 |
| 3:TRI vs. 28:Base | <0.0001 |
| 3:TRI vs. 28:TRI | <0.0001 |
| 4:Base vs. 4:TRI | >0.9999 |
| 4:Base vs. 7:Base | 0.0002 |
| 4:Base vs. 7:TRI | 0.0002 |
| 4:Base vs. 11:Base | 0.0013 |
| 4:Base vs. 11:TRI | 0.3344 |
| 4:Base vs. 14:Base | <0.0001 |
| 4:Base vs. 14:TRI | 0.0013 |
| 4:Base vs. 28:Base | <0.0001 |
| 4:Base vs. 28:TRI | <0.0001 |
| 4:TRI vs. 7:Base | 0.0002 |
| 4:TRI vs. 7:TRI | 0.0002 |
| 4:TRI vs. 11:Base | 0.0013 |
| 4:TRI vs. 11:TRI | 0.3344 |
| 4:TRI vs. 14:Base | <0.0001 |
| 4:TRI vs. 14:TRI | 0.0013 |
| 4:TRI vs. 28:Base | <0.0001 |
| 4:TRI vs. 28:TRI | <0.0001 |
| 7:Base vs. 7:TRI | >0.9999 |
| 7:Base vs. 11:Base | <0.0001 |
| 7:Base vs. 11:TRI | 0.2439 |
| 7:Base vs. 14:Base | <0.0001 |
| 7:Base vs. 14:TRI | <0.0001 |
| 7:Base vs. 28:Base | <0.0001 |
| 7:Base vs. 28:TRI | <0.0001 |
| 7:TRI vs. 11:Base | <0.0001 |
| 7:TRI vs. 11:TRI | 0.2439 |
| 7:TRI vs. 14:Base | <0.0001 |
| 7:TRI vs. 14:TRI | <0.0001 |
| 7:TRI vs. 28:Base | <0.0001 |
| 7:TRI vs. 28:TRI | <0.0001 |
| 11:Base vs. 11:TRI | <0.0001 |
| 11:Base vs. 14:Base | <0.0001 |
| 11:Base vs. 14:TRI | >0.9999 |
| 11:Base vs. 28:Base | <0.0001 |
| 11:Base vs. 28:TRI | <0.0001 |
| 11:TRI vs. 14:Base | <0.0001 |
| 11:TRI vs. 14:TRI | <0.0001 |
| 11:TRI vs. 28:Base | <0.0001 |
| 11:TRI vs. 28:TRI | <0.0001 |
| 14:Base vs. 14:TRI | <0.0001 |
| 14:Base vs. 28:Base | 0.0039 |
| 14:Base vs. 28:TRI | 0.0039 |
| 14:TRI vs. 28:Base | <0.0001 |
| 14:TRI vs. 28:TRI | <0.0001 |
| 28:Base vs. 28:TRI | >0.9999 |

**Table S5:** P-values obtained from PDI comparison between Base (SM-102) LNPs and 50% TRI-substituted LNPs over 28 days. Statistical differences were determined by two-sided, two-way ANOVA with *post hoc* Tukey’s comparison.

| Tukey’s multiple comparisons test | Adjusted P-value |
| --- | --- |
| 1:Base vs. 1:TRI | >0.9999 |
| 1:Base vs. 2:Base | 0.9577 |
| 1:Base vs. 2:TRI | 0.9933 |
| 1:Base vs. 3:Base | 0.9998 |
| 1:Base vs. 3:TRI | >0.9999 |
| 1:Base vs. 4:Base | >0.9999 |
| 1:Base vs. 4:TRI | >0.9999 |
| 1:Base vs. 7:Base | >0.9999 |
| 1:Base vs. 7:TRI | >0.9999 |
| 1:Base vs. 11:Base | 0.0016 |
| 1:Base vs. 11:TRI | 0.6109 |
| 1:Base vs. 14:Base | 0.0892 |
| 1:Base vs. 14:TRI | 0.9908 |
| 1:Base vs. 28:Base | <0.0001 |
| 1:Base vs. 28:TRI | <0.0001 |
| 1:TRI vs. 2:Base | 0.6354 |
| 1:TRI vs. 2:TRI | >0.9999 |
| 1:TRI vs. 3:Base | 0.9536 |
| 1:TRI vs. 3:TRI | >0.9999 |
| 1:TRI vs. 4:Base | 0.9890 |
| 1:TRI vs. 4:TRI | >0.9999 |
| 1:TRI vs. 7:Base | >0.9999 |
| 1:TRI vs. 7:TRI | >0.9999 |
| 1:TRI vs. 11:Base | 0.0002 |
| 1:TRI vs. 11:TRI | 0.2186 |
| 1:TRI vs. 14:Base | 0.0171 |
| 1:TRI vs. 14:TRI | 0.7931 |
| 1:TRI vs. 28:Base | <0.0001 |
| 1:TRI vs. 28:TRI | <0.0001 |
| 2:Base vs. 2:TRI | 0.2733 |
| 2:Base vs. 3:Base | >0.9999 |
| 2:Base vs. 3:TRI | 0.6518 |
| 2:Base vs. 4:Base | 0.9998 |
| 2:Base vs. 4:TRI | 0.9653 |
| 2:Base vs. 7:Base | 0.9676 |
| 2:Base vs. 7:TRI | 0.6187 |
| 2:Base vs. 11:Base | 0.0950 |
| 2:Base vs. 11:TRI | >0.9999 |
| 2:Base vs. 14:Base | 0.8755 |
| 2:Base vs. 14:TRI | >0.9999 |
| 2:Base vs. 28:Base | <0.0001 |
| 2:Base vs. 28:TRI | <0.0001 |
| 2:TRI vs. 3:Base | 0.6792 |
| 2:TRI vs. 3:TRI | >0.9999 |
| 2:TRI vs. 4:Base | 0.8255 |
| 2:TRI vs. 4:TRI | 0.9911 |
| 2:TRI vs. 7:Base | 0.9903 |
| 2:TRI vs. 7:TRI | >0.9999 |
| 2:TRI vs. 11:Base | <0.0001 |
| 2:TRI vs. 11:TRI | 0.0617 |
| 2:TRI vs. 14:Base | 0.0035 |
| 2:TRI vs. 14:TRI | 0.4131 |
| 2:TRI vs. 28:Base | <0.0001 |
| 2:TRI vs. 28:TRI | <0.0001 |
| 3:Base vs. 3:TRI | 0.9589 |
| 3:Base vs. 4:Base | >0.9999 |
| 3:Base vs. 4:TRI | 0.9999 |
| 3:Base vs. 7:Base | >0.9999 |
| 3:Base vs. 7:TRI | 0.9477 |
| 3:Base vs. 11:Base | 0.0193 |
| 3:Base vs. 11:TRI | 0.9861 |
| 3:Base vs. 14:Base | 0.4760 |
| 3:Base vs. 14:TRI | >0.9999 |
| 3:Base vs. 28:Base | <0.0001 |
| 3:Base vs. 28:TRI | <0.0001 |
| 3:TRI vs. 4:Base | 0.9908 |
| 3:TRI vs. 4:TRI | >0.9999 |
| 3:TRI vs. 7:Base | >0.9999 |
| 3:TRI vs. 7:TRI | >0.9999 |
| 3:TRI vs. 11:Base | 0.0003 |
| 3:TRI vs. 11:TRI | 0.2290 |
| 3:TRI vs. 14:Base | 0.0182 |
| 3:TRI vs. 14:TRI | 0.8068 |
| 3:TRI vs. 28:Base | <0.0001 |
| 3:TRI vs. 28:TRI | <0.0001 |
| 4:Base vs. 4:TRI | >0.9999 |
| 4:Base vs. 7:Base | >0.9999 |
| 4:Base vs. 7:TRI | 0.9870 |
| 4:Base vs. 11:Base | 0.0101 |
| 4:Base vs. 11:TRI | 0.9451 |
| 4:Base vs. 14:Base | 0.3276 |
| 4:Base vs. 14:TRI | >0.9999 |
| 4:Base vs. 28:Base | <0.0001 |
| 4:Base vs. 28:TRI | <0.0001 |
| 4:TRI vs. 7:Base | >0.9999 |
| 4:TRI vs. 7:TRI | >0.9999 |
| 4:TRI vs. 11:Base | 0.0018 |
| 4:TRI vs. 11:TRI | 0.6366 |
| 4:TRI vs. 14:Base | 0.0971 |
| 4:TRI vs. 14:TRI | 0.9931 |
| 4:TRI vs. 28:Base | <0.0001 |
| 4:TRI vs. 28:TRI | <0.0001 |
| 7:Base vs. 7:TRI | >0.9999 |
| 7:Base vs. 11:Base | 0.0019 |
| 7:Base vs. 11:TRI | 0.6451 |
| 7:Base vs. 14:Base | 0.0999 |
| 7:Base vs. 14:TRI | 0.9937 |
| 7:Base vs. 28:Base | <0.0001 |
| 7:Base vs. 28:TRI | <0.0001 |
| 7:TRI vs. 11:Base | 0.0002 |
| 7:TRI vs. 11:TRI | 0.2084 |
| 7:TRI vs. 14:Base | 0.0160 |
| 7:TRI vs. 14:TRI | 0.7790 |
| 7:TRI vs. 28:Base | <0.0001 |
| 7:TRI vs. 28:TRI | <0.0001 |
| 11:Base vs. 11:TRI | 0.3743 |
| 11:Base vs. 14:Base | 0.9634 |
| 11:Base vs. 14:TRI | 0.0530 |
| 11:Base vs. 28:Base | 0.1356 |
| 11:Base vs. 28:TRI | 0.1356 |
| 11:TRI vs. 14:Base | 0.9983 |
| 11:TRI vs. 14:TRI | 0.9996 |
| 11:TRI vs. 28:Base | 0.0001 |
| 11:TRI vs. 28:TRI | 0.0001 |
| 14:Base vs. 14:TRI | 0.7421 |
| 14:Base vs. 28:Base | 0.0028 |
| 14:Base vs. 28:TRI | 0.0028 |
| 14:TRI vs. 28:Base | <0.0001 |
| 14:TRI vs. 28:TRI | <0.0001 |
| 28:Base vs. 28:TRI | >0.9999 |

**Table S6:** P-values obtained from mRNA encapsulation efficiency comparison between Base (SM-102) LNPs and 50% TRI-substituted LNPs over 28 days. Statistical differences were determined by two-sided, two-way ANOVA with *post hoc* Tukey’s comparison.

| Tukey's multiple comparisons test | Adjusted P-Value |
| --- | --- |
| 1:Base vs. 1:TRI | <0.0001 |
| 1:Base vs. 2:Base | <0.0001 |
| 1:Base vs. 2:TRI | 0.0011 |
| 1:Base vs. 3:Base | >0.9999 |
| 1:Base vs. 3:TRI | <0.0001 |
| 1:Base vs. 4:Base | 0.1481 |
| 1:Base vs. 4:TRI | 0.0360 |
| 1:Base vs. 7:Base | 0.9494 |
| 1:Base vs. 7:TRI | <0.0001 |
| 1:Base vs. 11:Base | <0.0001 |
| 1:Base vs. 11:TRI | <0.0001 |
| 1:Base vs. 14:Base | <0.0001 |
| 1:Base vs. 14:TRI | <0.0001 |
| 1:Base vs. 28:Base | 0.0002 |
| 1:Base vs. 28:TRI | 0.0017 |
| 1:TRI vs. 2:Base | <0.0001 |
| 1:TRI vs. 2:TRI | <0.0001 |
| 1:TRI vs. 3:Base | <0.0001 |
| 1:TRI vs. 3:TRI | <0.0001 |
| 1:TRI vs. 4:Base | <0.0001 |
| 1:TRI vs. 4:TRI | <0.0001 |
| 1:TRI vs. 7:Base | <0.0001 |
| 1:TRI vs. 7:TRI | <0.0001 |
| 1:TRI vs. 11:Base | 0.9925 |
| 1:TRI vs. 11:TRI | <0.0001 |
| 1:TRI vs. 14:Base | 0.8137 |
| 1:TRI vs. 14:TRI | <0.0001 |
| 1:TRI vs. 28:Base | <0.0001 |
| 1:TRI vs. 28:TRI | <0.0001 |
| 2:Base vs. 2:TRI | 0.0004 |
| 2:Base vs. 3:Base | <0.0001 |
| 2:Base vs. 3:TRI | <0.0001 |
| 2:Base vs. 4:Base | <0.0001 |
| 2:Base vs. 4:TRI | <0.0001 |
| 2:Base vs. 7:Base | <0.0001 |
| 2:Base vs. 7:TRI | <0.0001 |
| 2:Base vs. 11:Base | <0.0001 |
| 2:Base vs. 11:TRI | <0.0001 |
| 2:Base vs. 14:Base | <0.0001 |
| 2:Base vs. 14:TRI | <0.0001 |
| 2:Base vs. 28:Base | <0.0001 |
| 2:Base vs. 28:TRI | <0.0001 |
| 2:TRI vs. 3:Base | 0.0002 |
| 2:TRI vs. 3:TRI | <0.0001 |
| 2:TRI vs. 4:Base | 0.9158 |
| 2:TRI vs. 4:TRI | <0.0001 |
| 2:TRI vs. 7:Base | 0.1142 |
| 2:TRI vs. 7:TRI | <0.0001 |
| 2:TRI vs. 11:Base | <0.0001 |
| 2:TRI vs. 11:TRI | <0.0001 |
| 2:TRI vs. 14:Base | <0.0001 |
| 2:TRI vs. 14:TRI | <0.0001 |
| 2:TRI vs. 28:Base | <0.0001 |
| 2:TRI vs. 28:TRI | <0.0001 |
| 3:Base vs. 3:TRI | <0.0001 |
| 3:Base vs. 4:Base | 0.0455 |
| 3:Base vs. 4:TRI | 0.1216 |
| 3:Base vs. 7:Base | 0.7295 |
| 3:Base vs. 7:TRI | <0.0001 |
| 3:Base vs. 11:Base | <0.0001 |
| 3:Base vs. 11:TRI | <0.0001 |
| 3:Base vs. 14:Base | <0.0001 |
| 3:Base vs. 14:TRI | <0.0001 |
| 3:Base vs. 28:Base | 0.0008 |
| 3:Base vs. 28:TRI | 0.0077 |
| 3:TRI vs. 4:Base | <0.0001 |
| 3:TRI vs. 4:TRI | 0.1231 |
| 3:TRI vs. 7:Base | <0.0001 |
| 3:TRI vs. 7:TRI | >0.9999 |
| 3:TRI vs. 11:Base | 0.0002 |
| 3:TRI vs. 11:TRI | <0.0001 |
| 3:TRI vs. 14:Base | <0.0001 |
| 3:TRI vs. 14:TRI | <0.0001 |
| 3:TRI vs. 28:Base | 0.9805 |
| 3:TRI vs. 28:TRI | 0.6852 |
| 4:Base vs. 4:TRI | <0.0001 |
| 4:Base vs. 7:Base | 0.9748 |
| 4:Base vs. 7:TRI | <0.0001 |
| 4:Base vs. 11:Base | <0.0001 |
| 4:Base vs. 11:TRI | <0.0001 |
| 4:Base vs. 14:Base | <0.0001 |
| 4:Base vs. 14:TRI | <0.0001 |
| 4:Base vs. 28:Base | <0.0001 |
| 4:Base vs. 28:TRI | <0.0001 |
| 4:TRI vs. 7:Base | 0.0002 |
| 4:TRI vs. 7:TRI | 0.0235 |
| 4:TRI vs. 11:Base | <0.0001 |
| 4:TRI vs. 11:TRI | <0.0001 |
| 4:TRI vs. 14:Base | <0.0001 |
| 4:TRI vs. 14:TRI | <0.0001 |
| 4:TRI vs. 28:Base | 0.9123 |
| 4:TRI vs. 28:TRI | 0.9995 |
| 7:Base vs. 7:TRI | <0.0001 |
| 7:Base vs. 11:Base | <0.0001 |
| 7:Base vs. 11:TRI | <0.0001 |
| 7:Base vs. 14:Base | <0.0001 |
| 7:Base vs. 14:TRI | <0.0001 |
| 7:Base vs. 28:Base | <0.0001 |
| 7:Base vs. 28:TRI | <0.0001 |
| 7:TRI vs. 11:Base | 0.0019 |
| 7:TRI vs. 11:TRI | <0.0001 |
| 7:TRI vs. 14:Base | <0.0001 |
| 7:TRI vs. 14:TRI | <0.0001 |
| 7:TRI vs. 28:Base | 0.7387 |
| 7:TRI vs. 28:TRI | 0.2702 |
| 11:Base vs. 11:TRI | <0.0001 |
| 11:Base vs. 14:Base | 0.1285 |
| 11:Base vs. 14:TRI | <0.0001 |
| 11:Base vs. 28:Base | <0.0001 |
| 11:Base vs. 28:TRI | <0.0001 |
| 11:TRI vs. 14:Base | <0.0001 |
| 11:TRI vs. 14:TRI | <0.0001 |
| 11:TRI vs. 28:Base | <0.0001 |
| 11:TRI vs. 28:TRI | <0.0001 |
| 14:Base vs. 14:TRI | <0.0001 |
| 14:Base vs. 28:Base | <0.0001 |
| 14:Base vs. 28:TRI | <0.0001 |
| 14:TRI vs. 28:Base | <0.0001 |
| 14:TRI vs. 28:TRI | <0.0001 |
| 28:Base vs. 28:TRI | >0.9999 |

**Table S7:** Size and polydispersity of ALC-0315 LNPs with 0%, 30%, 50% and 80% TRI substitution for cholesterol.

| LNP | Diameter (nm) | Polydispersity Index (PDI) |
| --- | --- | --- |
| 0% | 109.2 ± 34.5 | 0.1 ± 0.02 |
| 30% | 91.6 ± 23.9 | 0.07 ± 0.03 |
| 50% | 96.4 ± 31.5 | 0.11 ± 0.03 |
| 80% | 96.0 ± 44.6 | 0.22 ± 0.03 |

**Table S8:** Size and polydispersity of DLin-MC3-DMA LNPs with 0%, 30%, 50% and 80% TRI substitution for cholesterol.

| LNP | Diameter (nm) | Polydispersity Index (PDI) |
| --- | --- | --- |
| 0% | 77.1 ± 41.1 | 0.28 ± 0.04 |
| 30% | 63.0 ± 25.2 | 0.16 ± 0.03 |
| 50% | 76.3 ± 31.6 | 0.17 ± 0.01 |
| 80% | 63.5 ± 23.9 | 0.14 ± 0.01 |
